# In vitro modelling of human proprioceptive sensory neurons in the neuromuscular system

**DOI:** 10.1038/s41598-022-23565-3

**Published:** 2022-12-09

**Authors:** Maider Badiola-Mateos, Tatsuya Osaki, Roger Dale Kamm, Josep Samitier

**Affiliations:** 1 Institute for Bioengineering of Catalonia (IBEC)—Barcelona Institute of Science and Technology, 08028 Barcelona, Spain; 2 Department of Electronic and Biomedical Engineering, Universitat de Barcelona, 08028 Barcelona, Spain; 3 Centro de Investigación Biomédica en Red (CIBER-BBN), 28029 Madrid, Spain; 4 Department of Biological Engineering, Massachusetts Institute of Technology (MIT), 500 Technology Square, MIT Building, Cambridge, MA 02139 USA; 5 Department of Mechanical Engineering, Massachusetts Institute of Technology, 500 Technology Square, MIT Building, Cambridge, MA 02139 USA; 6 Present Address: The BioRobotics Institute, Department of Excellence in Robotics and AI, Scuola Superiore Sant’Anna, 56127 Pisa, Italy; 7 Present Address: Institute of Industrial Science, The University of Tokyo, 4-6-1, Komaba, Meguro-Ku, Tokyo, 153-8505 Japan

**Keywords:** Biological techniques, Biotechnology, Cell biology, Neuroscience

## Abstract

Proprioceptive sensory neurons (pSN) are an essential and undervalued part of the neuromuscular circuit. A protocol to differentiate healthy and amyotrophic lateral sclerosis (ALS) human neural stem cells (hNSC) into pSN, and their comparison with the motor neuron (MN) differentiation process from the same hNSC sources, facilitated the development of in vitro co-culture platforms. The obtained pSN spheroids cultured interact with human skeletal myocytes showing the formation of annulospiral wrapping-like structures between TrkC + neurons and a multinucleated muscle fibre, presenting synaptic bouton-like structures in the contact point. The comparative analysis of the genetic profile performed in healthy and sporadic ALS hNSC differentiated to pSN suggested that basal levels of ETV1, critical for motor feedback from pSN, were much lower for ALS samples and that the differences between healthy and ALS samples, suggest the involvement of pSN in ALS pathology development and progression.

## Introduction

**Figure 1:**
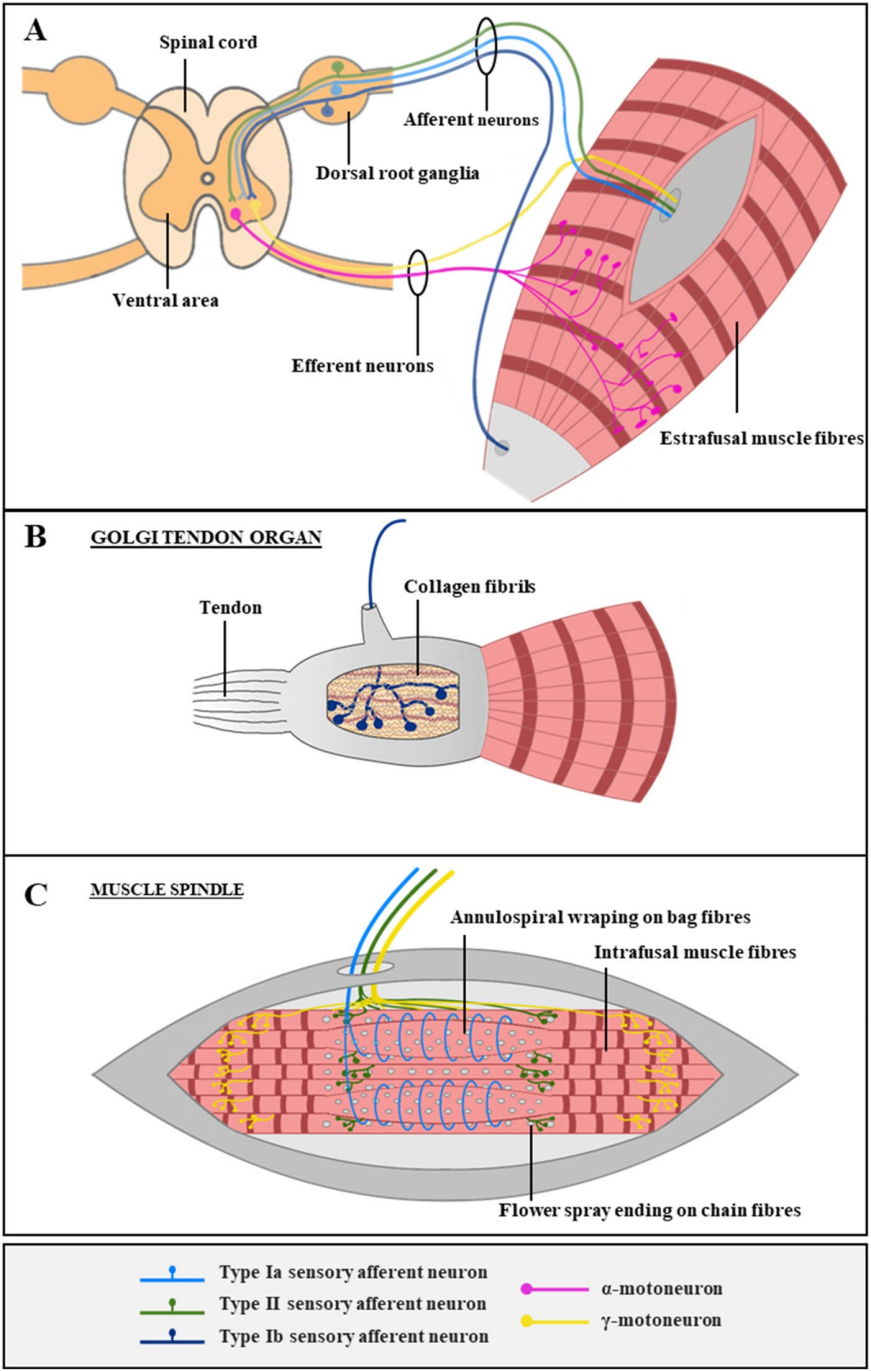
Spinal neuromuscular circuit and proprioceptive receptors in the muscle. (**A**) Efferent neurons in the muscle include the innervation of extrafusal muscle fibres by α-MN (pink), triggering the contraction, and the innervation of intrafusal muscle fibres by γ-MN (yellow), modulating the excitability input and the contractibility of the muscle-spindle. The somas of α-MN and γ-MN are located in the ventral area of spinal cord, and they extend long axons towards skeletal muscle. Afferent neurons in the muscle include types Ia (clear blue), II (green) and Ib (dark blue) afferents, that are in charge of proprioception through specialised organs: Golgi tendon organ (GTO) and muscle spindles. They sense muscle contraction and send the signal back to the spinal cord, passing through dorsal root ganglia, where their somas are located. (**B**) Golgi tendon organ, located at the joint between muscle fibres and tendons, is formed by encapsulated structures of collagen fibrils that compress when the muscle is contracted, innervating a single Ib afferent neuron. (**C**) Muscle spindles, located inside extrafusal muscle fibres, are formed by encapsulated intrafusal muscle fibres (bag and chain fibres) connected to Ia and II afferent sensory endings. Type Ia sensory fibres form annulospiral wrappings and sense the rate of length change. Type II sensory fibres form flower-spray endings and sense relative muscle length. γ-motoneurons innervate polar regions of intrafusal muscle fibres and calibrate the sensitivity of muscle spindles, forming the fusimotor system.

The spinal neuromuscular circuit and neuromuscular diseases (NMD) have been thoroughly studied for years paying special attention to the α-motoneuron and its connection with skeletal muscle (SkM) cells^1^. In addition the role of other cells as Schwann cells^2–6^, astrocytes^7^, microglia^8,9^, oligodendrocytes^10,11^, interneurons^12^, and γ-MN^13^, has been considered. However the role of proprioceptive sensory neurons, and their functional interactions has been underestimated^14^. Proprioceptive sensory neurons (pSN), or sensorimotor afferents, represent 7.5% of DRG neurons^15^. Sensorimotor proprioceptive afferents connect to specialized proprioceptive receptors (muscle spindle and Golgi tendon organ), and send information about muscle contraction status to the spinal cord, modulating MN activity (see Fig. 1). Muscle spindles are located inside mammalian skeletal muscle, in muscle fascicles of peripheral nerves and their function is to sense changes in muscle length of the specific muscle they innervate^16–19^. Sensorimotor afferents type Ia wrap around the central region of bag_1_ and other intrafusal muscle fibres forming a structure called annulospiral wrapping (ASW). Type II afferents contact with bag_2_ and chain intrafusal muscle fibres in areas adjacent to the central region forming a flower spray ending (FSE) structure. The muscle spindle excitatory input is modulated by γ-motoneurons and β-motoneurons that innervate the muscle spindle on the polar regions of intrafusal muscle fibres forming the fusimotor system^19–21^. Failures in any part of this circuit can hamper muscle movement, and interfere with reflexes, causing many sensorimotor neuromuscular diseases as amyotrophic lateral sclerosis (ALS). ALS is a neuromuscular disease with a worldwide yearly mortality rate of 30.000 patients^22^, with an incidence of 2–3 per 100.000 individuals in Europe^23^, is usually fatal within 2–5 years^24^. Despite considerable effort, its pathology is still poorly understood^22^. Although ALS has long been known as a motor neuron disease, it has broader ramifications.

Proprioceptive sensory neuron degeneration, altered morphology, signal transmission and spindle malfunctions in ALS have each been observed in ALS mouse models^13,25–28^. There are, however, few published clinical studies that clearly demonstrate alterations in proprioception in human ALS patients^29,30^. And despite recent advances in ALS models, both in mouse models and in vitro studies using human cell lines^31,32^, and evidence supporting the hypothesis of an important role of pSN in the early stages of ALS, most studies continue to focus exclusively on motoneurons and their connexion to skeletal myocytes.

Human neuromuscular circuits can be studied in vitro owing to recent advances in stem cell technologies. Protocols to differentiate hNSC directly into motoneurons (MN) have been published^33^. For mechanoreceptive and nociceptive sensory neurons, there are various differentiation protocols^34^, and several studies have produced cell cultures enriched in all subtypes of sensory neurons, including proprioceptive sensory neurons (pSN)^15,35–43^. No validated protocol, however, yet exists to differentiate specifically pSN and characterise or detect them. Therefore, current protocols for differentiating hNSC into pSN involve several intermediate steps: differentiating first hNSC into neural crest stem cells^44–46^, then into PNS lineage, peripheral neurons, peripheral sensory neuron, to finally obtain pSN. The lack of consensus on pSN classification and a specific differentiation protocol for human cells comes together with the challenge of differentiating properly skeletal muscle cells and culturing both types of cells from human origin in vitro. There has only recently been published a protocol to differentiate intrafusal and extrafusal muscle fibres^47^. Furthermore, there is a small literature on experiments performing in vitro coculture of sensory neurons with skeletal muscle cells^43,48–50^, in contrast with the vast collection of studies on the neuromuscular motor pathway^1^. Also, while most of these publications focusing on the neuromuscular sensory afferents are performed with rodent tissue, so do not accurately mimic human conditions, they have helped elucidate the role of several molecules on proprioception and mechanotransduction. The limited collection of studies performing in vitro cell culture of SN-SkM connection in the last decade (summarised in Table S1 further illustrates our lack of knowledge and the need to examine it. Out of those studies, they all perform 2D cell culture of a healthy sensory afferent circuit^43,48–50^, and, only Guo et al*.*^43^ use human cells. To date there are no published in vitro studies of the proprioceptive pathway using 3D cell culture, integrating it with the motor pathway, or culturing cells into microfluidic compartmentalised devices.

The objectives of the present study were to develop a protocol for human pSN differentiation, to recreate pSN connections with SkM on a microfluidic chip, and to evaluate the differences of pSN in healthy and ALS disease cells. A comparative analysis of gene expression between healthy and ALS cells when exposed to different media conditions allowed to underline the impact of pSN in ALS. The results obtained suggest that the lower ETV1 expression could potentially have a key role in the functional connection degradation for pSN in ALS pathology.

## Results

**Figure 2:**
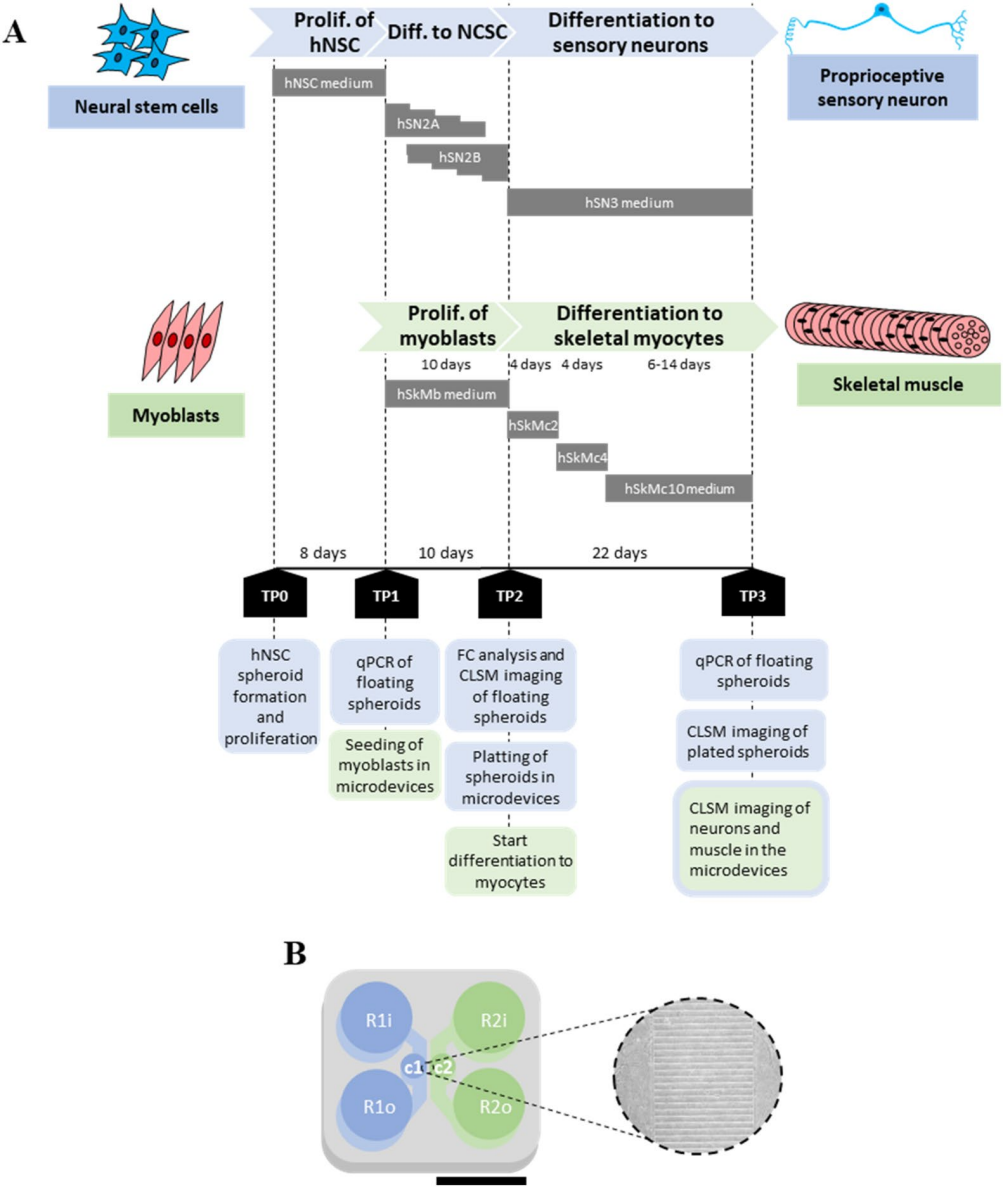
Diagram of the events and timepoints (TP) for the microfluidic experiments performed for the coculture of sensory neurons with skeletal muscle cells. (**A**) Diagram of the events, media utilised and timepoints (TP) for each cell type used. Neural stem cells (hNSC) were proliferated and then differentiated to neural crest stem cells and to sensory neurons, using four different media compositions. Human skeletal myoblasts were proliferated and differentiated to skeletal myocytes using four different subsequent media compositions. Several tests were conducted at different timepoints: flow cytometry (FC) analysis, confocal laser scanning microscopy (CLSM) imaging, and qPCR. (**B**) Layout of the Xona microfluidic device used. Xona microfluidcs SND900 device with a perforation in the left side (c1) for direct seeding of the SN spheroid and a perforation in the right side (c2) for the seeding of SkM; inlet and outlet reservoirs with medium for SN (R1i, R1o) and inlet and outlet reservoirs with medium for SkM (R2i, R2o). Augmented bright field image of the intersection formed by microchannels.

We assessed the effect of several differentiation protocols on pSN marker differences and selected the one optimal for inducing TrkC positive neurons. Human TrkC positive neurons were cultured together with human skeletal myoblasts differentiated to skeletal myocytes. The experiments conducted are summarised in Fig. 2, where the diagram of the events, media utilised and timepoints (TP) for each cell type used are defined.

### Characterisation of differentiated pSN

**Figure 3:**
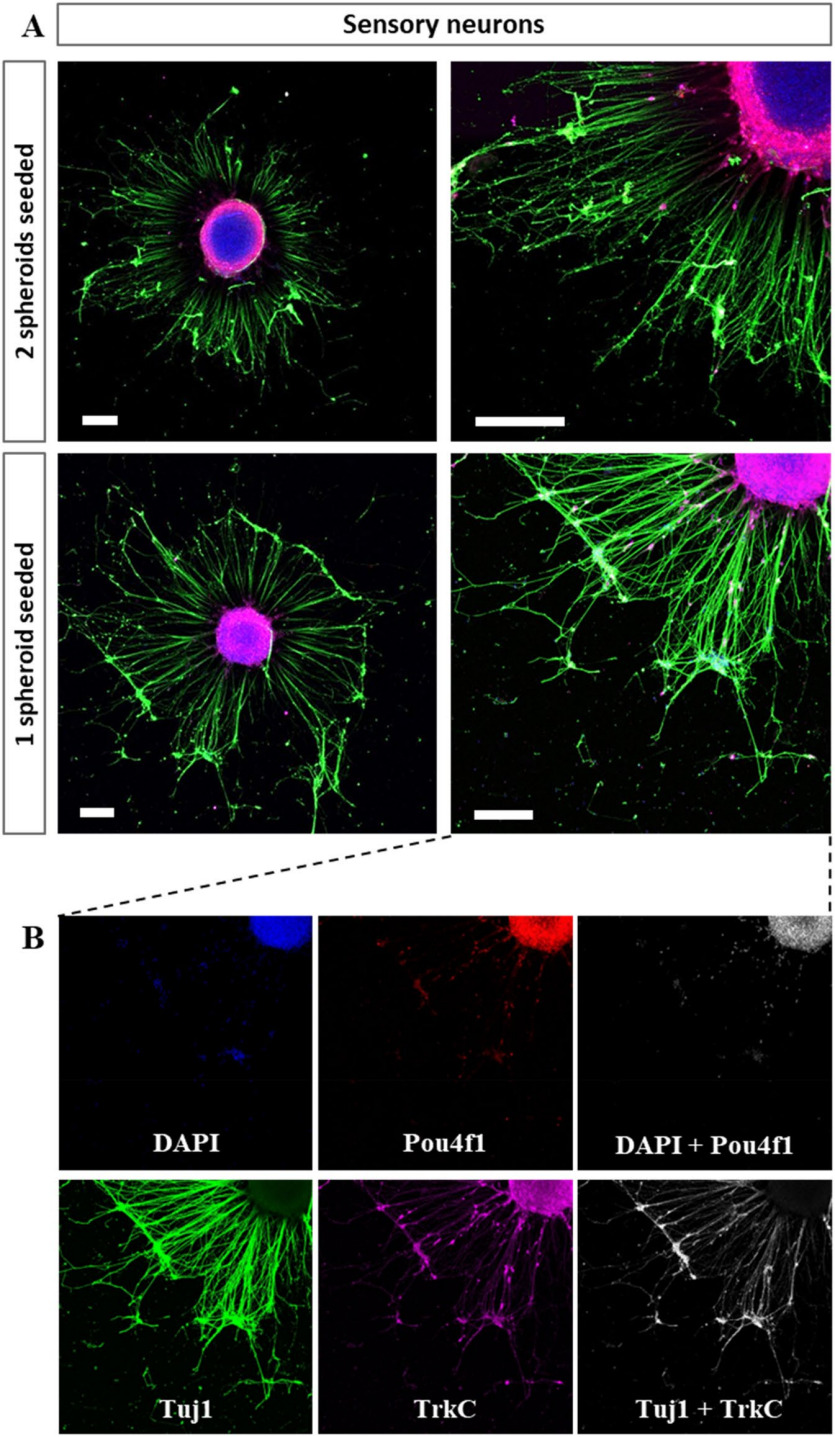
Immunostaining of healthy hNSC spheroids differentiated to SN. (**A**) Comparison between SN spheroids platted isolated or with another neighbouring spheroid onto Matrigel coated surfaces at TP2. Spheroids are differentiated through differentiation protocol A. Images taken at TP3. (**B**) Detailed image of an isolated spheroid. Nuclei are stained in blue, TUJ1 in green, Pou4f1 in grey and TrkC in magenta. Images showing only colocalising pixels (between DAPI and Pou4f1; and between Tuj1 and TrkC) are shown in greyscale. Scale bars are 200 µm.

The immunostaining images taken in healthy plated spheroids (Fig. 3B), showed few cells (only stained with DAPI, not positive to Tuj1, Pou4f1 or TrkC) located in the corona of the spheroid, high sensory neuron signal in the outside of the spheroid core (stained with Pou4f1), low number of neurites (stained with Tuj1) and low specificity in the differentiation towards pSN (low TrkC signal in the axons of the spheroid corona). The quantification of the images obtained revealed that an average of 10.6% (± 6.5 SD) of Tuj1 positive neurons were also TrkC positive, and an average of 22.2% (± 6.1 SD) of all nuclei stained with DAPI were positive for Pou4f1.

**Figure 4:**
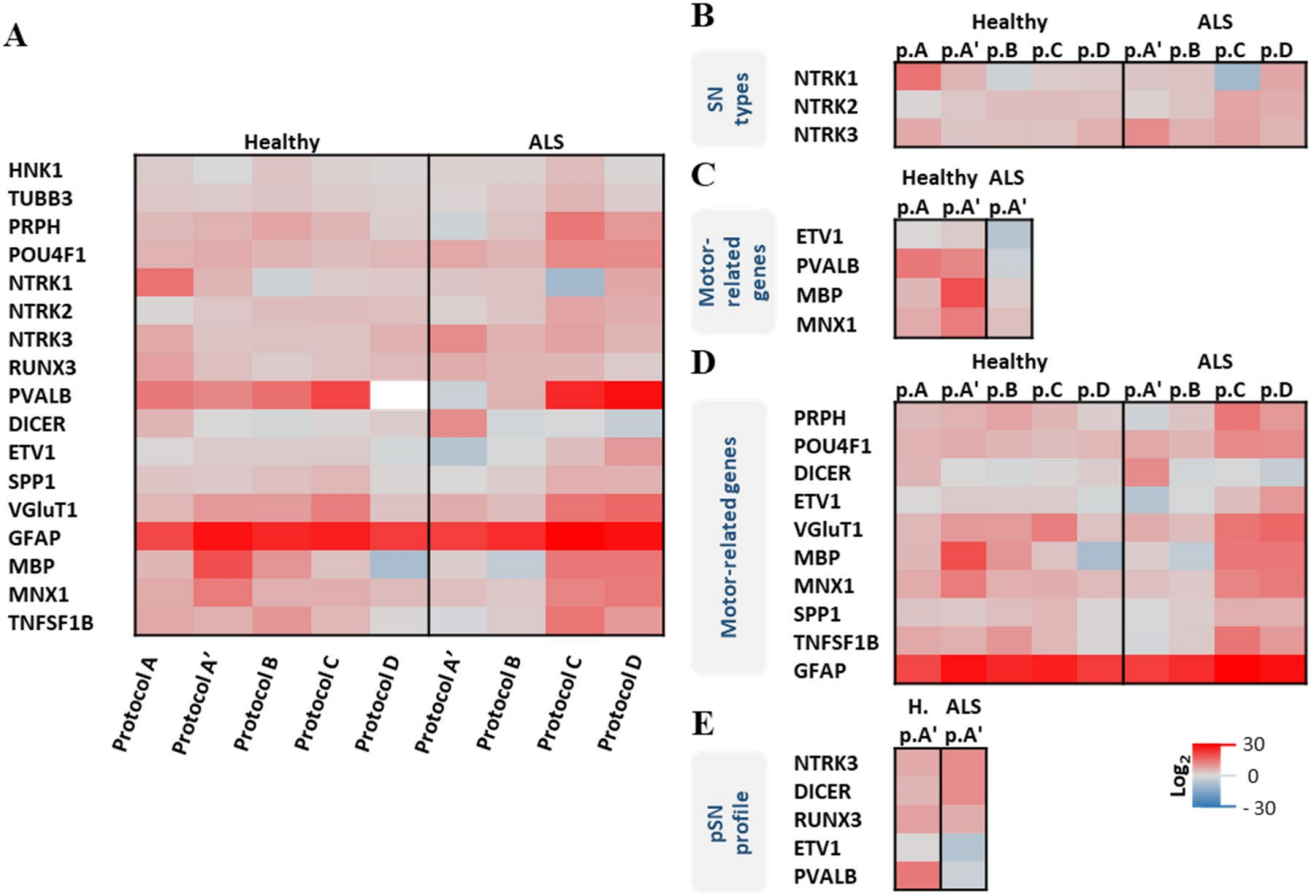
Characterisation of SN spheroids through qPCR. (**A**) qPCR normalised mean fold change results at TP3 of healthy and ALS SN spheroids undergoing differentiation *protocols A-D*, normalised against mRNA expression of SN spheroids at TP1. The expression of PVALB for healthy samples undergoing *protocol D* was undetectable. A collection of detailed highlights extracted from this analysis (**A**) is shown in figures (**B-E**). (**B**) SN type production analysis based on the typical three markers for nociceptive (NTRK1), mechanoreceptive (NTRK2) and proprioceptive (NTRK3) neurons. (**C**) Comparison of some motor-related genes levels in healthy and ALS. (**D**) Assessment of the effect of ROCK inhibitor Y27632 (protocols C&D) on some motor-related genes in healthy and ALS samples. (**E**) Comparison of basal levels of pSN genetic profile markers in healthy and ALS samples.

A detailed analysis revealed that the protocols without CHIR99021 and/or ROCK inhibitor Y27632 (*protocols A* and *A’*) were both able to induce higher levels of NTRK3 expression (Fig. 4B and Fig. S2), and therefore, the best to obtain a diverse population of neurons enriched in pSN. Healthy cells differentiated with *protocol A*, and ALS cells differentiated with *protocol A’* presented significantly less amplification cycles for NTRK3 and therefore higher genetic expression (Fig. S2).

### Characterisation of pSN differentiation at intermediate timepoint

The immunostaining and flow cytometry (FC) analysis performed onto floating spheroids of healthy and ALS cells undergoing SN differentiation through different protocols at TP2 showed that all samples assessed express HNK1 (an enzyme involved in cell metabolism and frequently used for the characterisation of neural crest stem cells^35,46,51^), both analysed through immunostaining and through FC, as seen in Fig. S4. The representative image of immunostaining of floating spheroids shows that cells present in the spheroid are successfully expressing HNK1 and there is a little expression of TUJ1. Results from FC analysis showed that all samples analysed were HNK1 positive, regardless of being 2D culture or spheroids of different seeding densities (Fig. S4B and Fig. S4A), healthy or ALS samples undergoing different differentiation protocols (Fig. S4C and Fig. S4B).

At intermediate timepoint, samples were evaluated through flow cytometry (FC) and immunostaining of floating spheroids. These results (Fig. S4) suggested an adequate differentiation towards neural crest stem cells with higher specificity with *protocol A*, as all samples assessed through immunostaining and FC expressed HNK1, regardless of being 2D cultures, spheroids of different seeding densities, healthy or ALS samples. To understand the influence of the seeding density and the spheroid on the initial differentiation, experiments were performed in 2D cell cultures and spheroids of healthy and ALS hNSC undergoing SN differentiation *protocol A*. These results showed that seeding density has little effect on the differentiation towards HNK1 + cells. The analysis performed in healthy and ALS hNSC spheroids undergoing the rest of differentiation protocols *(A’-D)* to assess the effects of each differentiation protocol onto the initial steps of the differentiation showed that all protocols were able to induce the formation of HNK1 + cells at TP2 in healthy and ALS hNSC.

### Comparison between SN and MN differentiation from hNSC

The transversal area quantification performed in hNSC spheroids undergoing differentiation towards SN and MN, showed that floating SN spheroids remain about the same size during their differentiation whereas MN increase up to four times (see Fig. S6). Although there are no significant differences between samples at each timepoint due to a low sample number (n = 3) (Fig. S6B), globally there is a trend of an augment in MN size among all timepoints, different from the trend among SN samples (**p-value < 0.05) (Fig. S6C). The BF images taken in plated spheroids undergoing differentiation to SN and MN, showed a clear augment of cells in the periphery of MN spheroids, contrary to SN spheroids, and migration of SN spheroids (Fig. S7). Moreover, it is observable in SN spheroids a tendency to approximate to each other, fusing both coronas at 15 ddiff. MN spheroids did not show any migration trend and their inter-spheroid distance was only affected by the spheroid core growth (Fig. S7).

**Figure 5:**
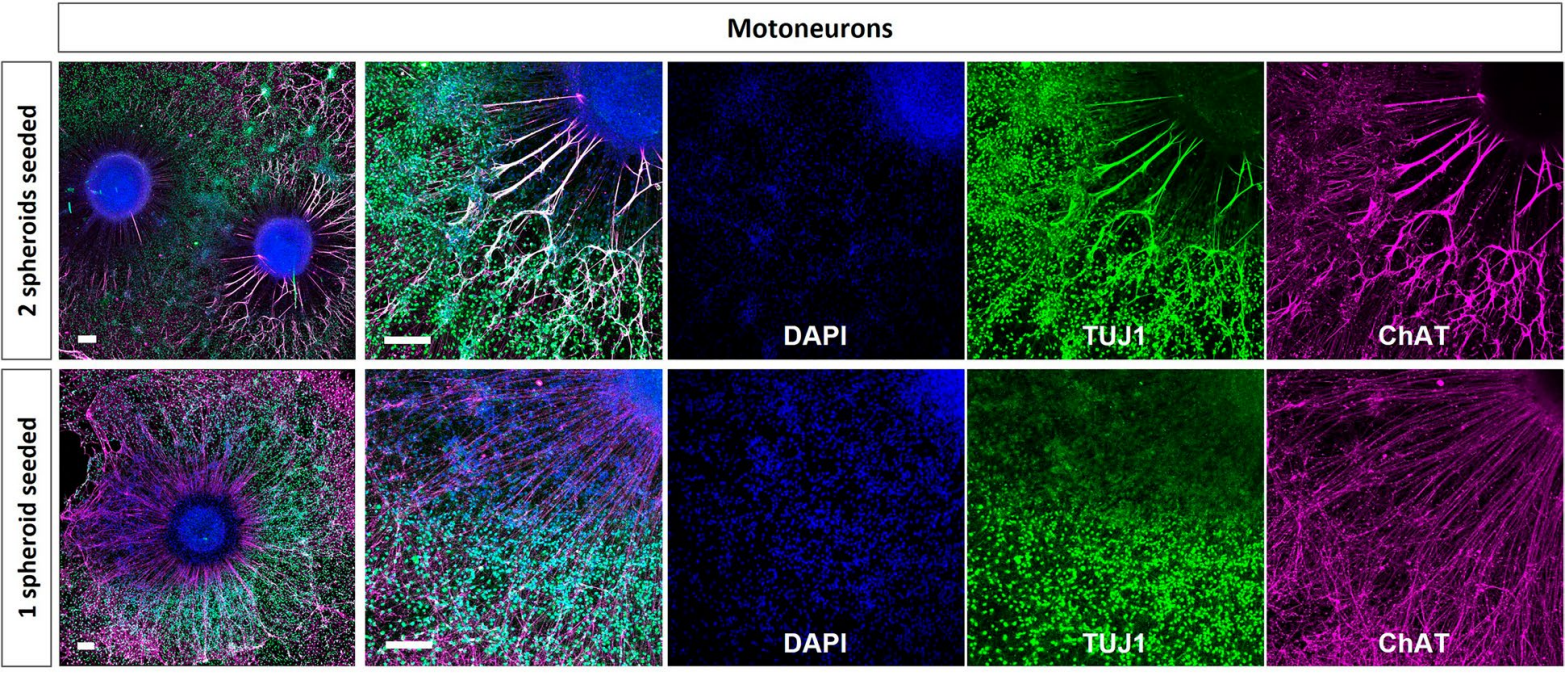
Immunostaining of healthy hNSC spheroids differentiated to SN. Comparison between MN spheroids platted isolated or with another neighbouring spheroid onto Matrigel coated surfaces at TP2**.** Images taken at TP3. Nuclei are stained in blue, TUJ1 in green, and ChAT in magenta. Scale bars are 200 µm.

The immunostaining images taken in plated spheroids undergoing *SN differentiation protocol* (Fig. 5, sensory neurons), showed few cells in the corona of the spheroid (stained with DAPI), high sensory neuron signal in the outside of the spheroid core (stained with Pou4f1), low number of neurites (stained with Tuj1) and low specificity in the differentiation towards pSN (low TrkC signal in the axons of the spheroid corona). Furthermore, when two spheroids were seeded together, they fused and formed a bigger one (Fig. 3A).

The immunostaining images taken in plated spheroids undergoing *MN differentiation protocol* (Fig. 5, motoneurons), showed lots of cells in the corona of the spheroid (stained with DAPI) and high specificity in the differentiation protocol (high ChAT signal). Contrary to SN spheroids, when two MN spheroids were seeded together, they did not migrate.

### Coculture of pSN with SkM cells

**Figure 6:**
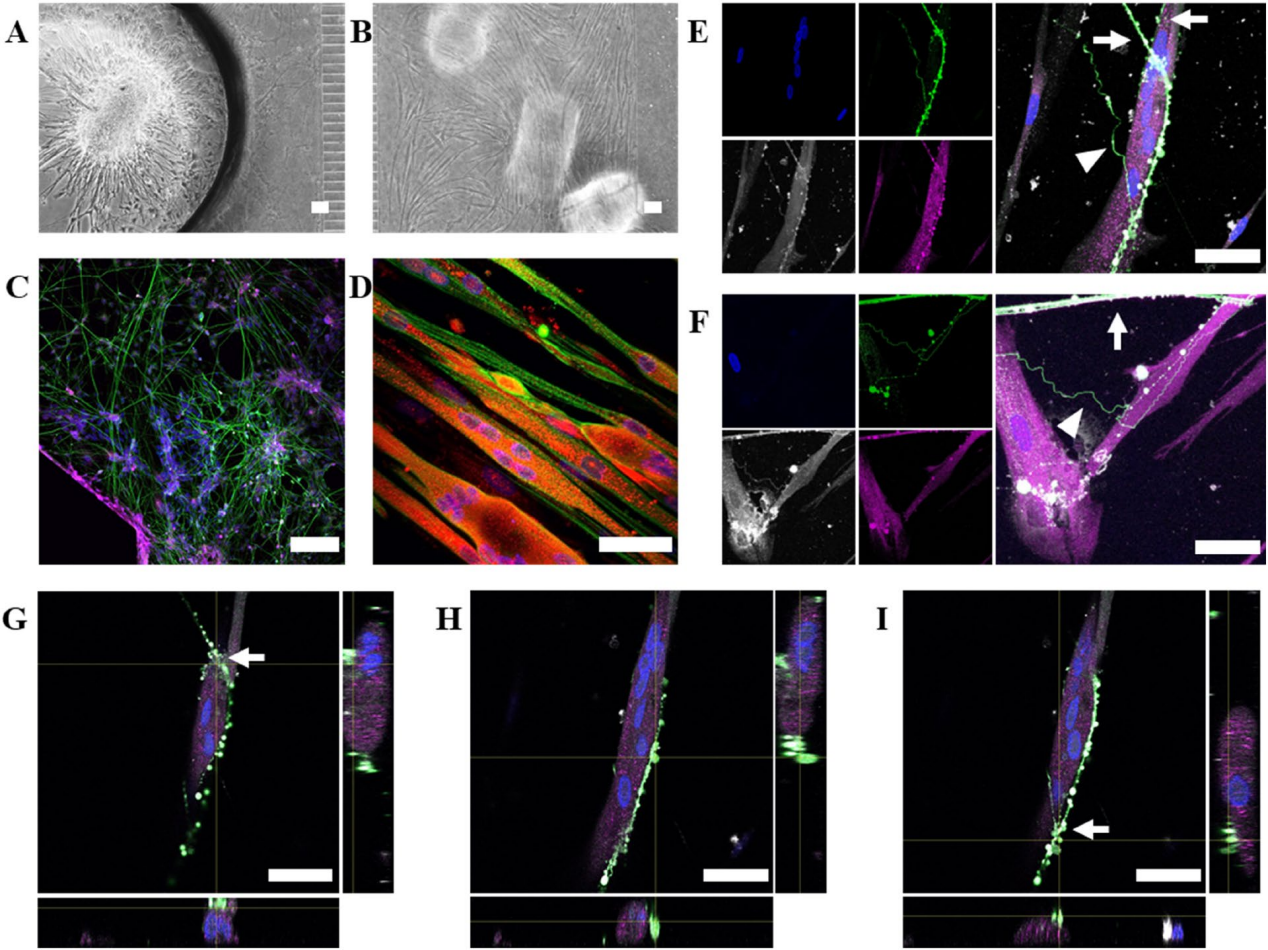
Compartmentalised coculture of healthy SN spheroids undergoing differentiation protocol A and SkM in Xona microfluidic devices. (**A**) Bright field image of SN compartment with the spheroid seeded in the hole near microchannels at TP3. (**B**) Bright field image of SkM compartment at TP2 + 12 DIV. (**C**) Immunostaining of the SN compartment at TP3 showing nuclei in blue, TUJ1 in green, and TrkC in magenta. (**D**) Immunostaining of the SkM compartment at TP3, showing nuclei in blue, MHC in red and α-actinin in green. Scale bar is 100 µm for figures (**A–D**). (**E**) Interaction between a TrkC + axon (arrow) and a TrkC- axon (triangle) with a multinuclear muscle fibre. (**F**) Interaction of a TrkC + axon (arrow) and one TrkC- axon (triangle) with another muscle fibre. For figures (**E–F**), nuclei are stained in blue, TUJ1 in green, phalloidin in grey and TrkC in magenta. Scale bars are 25 µm. (**G–I**) Orthogonal views of (**E**) showing the interaction between TrkC + neuron and a muscle fibre. Nuclei are shown in blue, TUJ1 in green, phalloidin in grey and TrkC in magenta. Different planes higher (left) or lower (right) show structures compatibles with synaptic buttons (indicated with an arrow). Scale bars are 25 µm.

**Figure 7:**
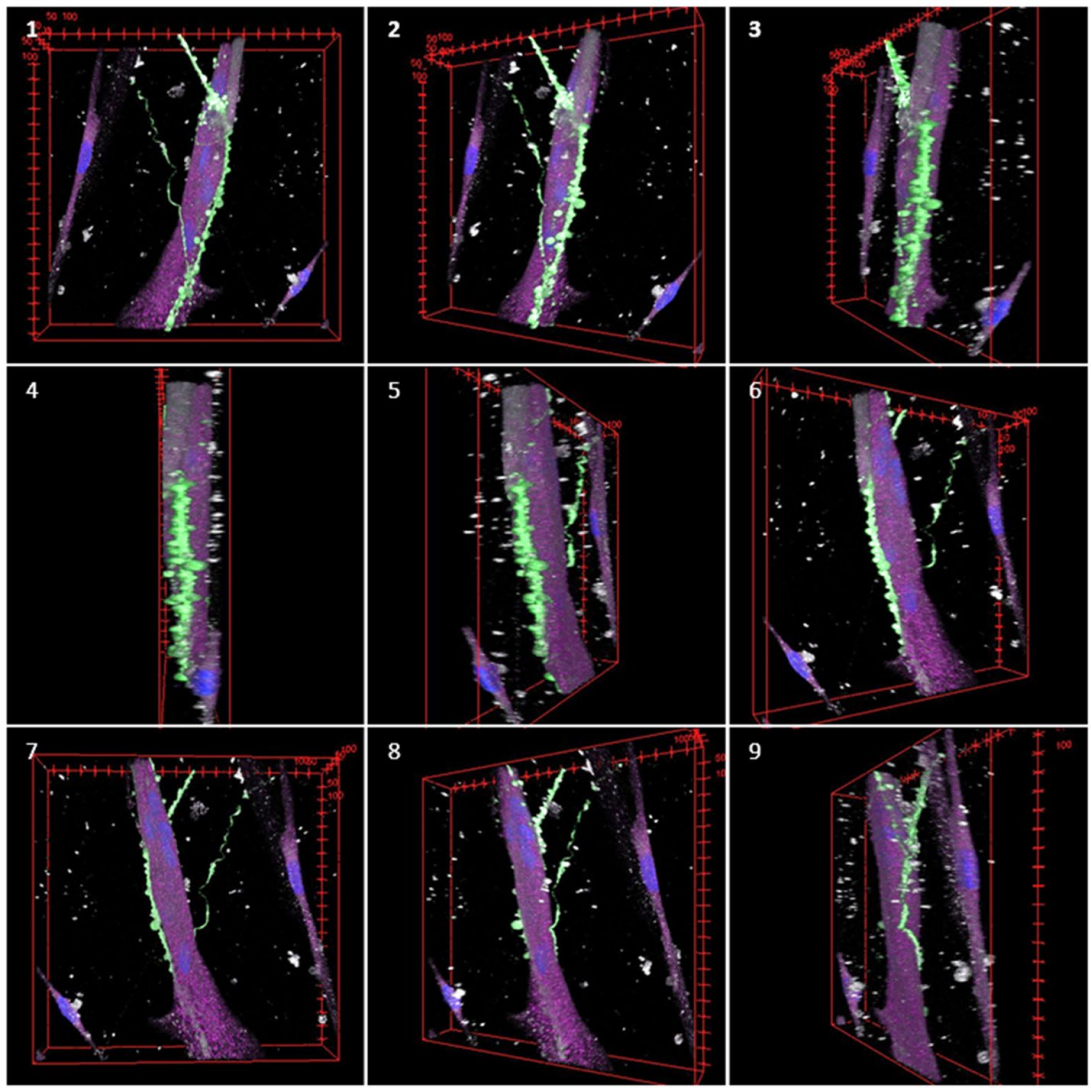
Montage of the different views of a 3D reconstruction of a confocal image of SN neural processes connecting with SkM rotating along its Y axis. This montage is a 360° rotation on Y axis, made from a 3D view reconstruction of Fig. 6E, shown in Movie S2. Nuclei are shown in blue, TUJ1 in green, phalloidin in grey and TrkC in magenta.

The compartmentalised coculture of SN spheroids with hSkMc in Xona microfluidic devices showed that SN spheroids (Fig. 6A) were able to extend their neurite projections throughout the whole neural compartment (Fig. 6C), expressing few TrkC + axons colocalising with Tuj1. The hSkMc with random alignment in the first steps of the differentiation (Fig. 6B) acquired aligned structure of their multinuclear fibres (Fig. 6D and Fig. S8). However, hSkMc fibres were not completely differentiated as MHC and α-actinin did not show the typical striated sarcomere organisation. Both TrkC + and TrkC- axons were found interacting with SkM fibres (Fig. 6E and F). We could also observe between TrkC positive neuron and a multinucleated muscle fibre with bag fibre morphology, a structure that resembles annulospiral wrapping-like configuration with the morphology of synaptic bouton-like structures in the contact points (Fig. 6E–I, Fig. 7, Fig. S9, Movie S1 and Movie S2).

### Analysis of the effect of differentiation protocols in healthy and ALS cells

Healthy and sporadic ALS-diseased hNSC differentiated to pSN showed diverse effects upon the exposure to the different components used for their differentiation. A panel of 20 genes was evaluated for qPCR experiments based on literature references for the expression of those genes (see Table S2). The increment of NT-3 concentration on the medium (*protocol A’* compared to *protocol A*, Fig. 4A and Fig. S2), induced in healthy cells a significant increase in the expression of MBP (p < 0.001), GFAP (p < 0.0001), and a significant decrease in the expression of DICER (p < 0.001), HNK1 (p < 0.05), NTRK3 (p < 0.001), and RUNX3 (p < 0.001).

The addition of CHIR99021 on the medium on top of the increment of NT-3 (*protocol B* compared to *protocol A’*, Fig. 4A and Fig. S2), induced in healthy cells a decrease in NTRK1 (p < 0.01) and MNX1 (p < 0.01), and a decrease in DICER (p < 0.05) in ALS samples.

The addition of ROCK inhibitor Y27632 on top of the increment of NT-3 on the medium (*protocol D* compared to *protocol A’*, Fig. 4A and Fig. S2), induced an opposite effect between healthy and ALS samples in PRPH, GFAP, MBP and MNX1, causing in all a decreased for healthy cells (p < 0.001, p < 0.05, p < 0.001 and p < 0.01 respectively) and an increase in ALS cells (p < 0.0001, p < 0.01, p < 0.001 and p < 0.001 respectively). Furthermore, it induced in ALS samples a decrease of DICER (p < 0.0001), RUNX3 (p < 0.0001) and NTRK3 (p < 0.01), together with an increase in NTRK2 (p < 0.05), vGluT1 (p < 0.01), SPP1 (p < 0.01) and ETV1 (p < 0.0001) and PVALB (p < 0.05).

The addition of ROCK inhibitor Y27632 and the addition of CHIR99021 on top of the increment of NT-3 on the medium (*protocol C* compared to *protocol A’*, Fig. 4A and Fig. S2), induced in healthy and ALS cells a decrease of NTRK1 (p < 0.05) and an increase in PVALB (p < 0.05). It induced a MBP increase in healthy cells (p < 0.0001) and a decrease in ALS ones (p < 0.001). Furthermore, in ALS samples, it induced an increase of TUBB3 (p < 0.05), PRPH (p < 0.001), NTRK2 (p < 0.0001), ETV1 (p < 0.0001), SPP1 (p < 0.01), GFAP (p < 0.0001), MNX1 (p < 0.01), TNFSF1B (p < 0.01), and a decrease in DICER (p < 0.05).

The analysis performed on healthy and ALS spheroids (Fig. 4A) revealed in general quite different expression patterns between healthy and ALS samples undergoing protocols containing ROCK inhibitor Y27632 in the medium (*protocols C* and *D*). In ALS cells, it induced a significant increase on the expression of some motor-system related genes—myelin (MPB +), peripheral neurons (PRPH +), motoneurons (MNX1 +), pSN functionality related markers (ETV1 and VGluT1), TNFSF1B and SPP1 (Fig. 4D and Fig. S2, *protocols C* and *D*)—and a decrease of DICER.

When comparing ALS cells to healthy cells, we see that exposing cells to the same condition (*protocol A’*), at TP3 ALS samples show a significantly lower expression of certain motor-related genes as compared to their relative healthy ones: ETV1, motor system cells (PVALB +), myelin (MBP +), and motoneurons (MNX1 +) (Fig. 4C and Fig. S1, *protocol A’*).

Similarly, when exposing samples to those same conditions (*protocol A’*), at TP3 we can observe inconsistencies in pSN genetic profile in healthy and ALS samples, showing in the ALS cells higher genetic expression levels of NTRK3 and DICER; lower levels in ETV1 and PVALB, and similar levels in RUNX3 expression (Fig. 4E and Fig. S1, *protocol A’*).

## Discussion

In ALS, α-MN forming the fast-fatigable motor units are the first to undergo degeneration, followed by fast fatigue resistant MN, whereas neighbouring slow α-MN, γ-MN, ocular MN and anal sphincter neuromuscular junctions (NMJ) remain resistant to degeneration^13,52,53^. The lack of stretch-reflex in the eye, external urethral and anal sphincters, and the lack of direct excitatory input from pSN (type Ia) onto γ-MN—both cases with resistance mechanisms to ALS—suggest together that stretch sensitivity of spindles and pSN feedback could be related to the pathogenesis and progression of ALS^13,54^. Ablation of the spindle has even been attributed to a decrease in MN death in ALS^13^, relating muscle spindle afferents to ALS early triggers of MN degeneration^55^. It is thought that α-MN and pSN may have similar molecular mechanisms involved in the failure, maintenance and repair occurring in ALS^27^. And although the degeneration occurring in pSN in ALS models exhibits different characteristics in each mutation^27^, it is not yet understood what drives the different responses.

In this study, we have compared the gene expression alterations of differentiated healthy and sporadic ALS human neural progenitors into pSN exposed to different media conditions and tried to recreate the connection between pSN and SkM in a microfluidic device. The differentiated healthy and ALS neural cells obtained without CHIR99021 and/or ROCK inhibitor Y27632 (*protocol A* and *A’*) generated a SN population with highest enrichment in pSN, being SN population predominantly shifted to nociceptive SN (NTRK1 +), with few mechanoreceptive SN (NTRK2 +) and some pSN (NTRK3 +) for healthy cells and predominantly pSN for ALS samples (see Fig. 4B and Fig. S1).These results were also observed in the immunostaining performed in healthy SN spheroids undergoing *protocol A* (Fig. 3B), where a diverse population of neurons was obtained, expressing 22.2% (± 6.1 SD) of the cells Pou4f1 in their soma—colocalising with DAPI—and 10.6% (± 6.5 SD) of Tuj1 positive neurons, TrkC in the whole cell body—colocalising with Tuj1 expression.

Usually, ALS is characterised by the downregulation of MBP^10,11^, and the upregulation of PRPH^56^, GFAP^57^ and TNFSF1B^58^. Nevertheless, DICER and SPP1 could also be involved in ALS considering that DICER is known to be downregulated in ataxia^59,60^, and SPP1 is known to be upregulated in inflammatory processes of dystrophic and injured muscles^61,62^. The target gene expression at the end of the differentiation in ALS cells versus healthy cells, when ROCK inhibitor Y27632 is added on the medium, (Fig. 4D and Fig. S2, *protocols C* and *D*), shows the upregulated expression of many genes in ALS samples, matching some ALS expression patterns. In the results obtained, we can observe in ALS samples undergoing protocols *C* and *D*, that peripherin and TNFSF1B levels are high, as expected for ALS^56,58^, DICER levels are lower (only in *protocol D*) as observed in ataxia^59,60^, and SPP1 is higher, as observed in inflammatory processes of dystrophic and injured muscles^61,62^. However, comparing to healthy cells, GFAP expression was not upregulated. Motoneurons marker (MNX1) and pSN functionality related markers (ETV1 and vGluT1) were also upregulated in ALS samples. VGluT1 is expressed at the end terminals of pSN, at the synaptic contacts of interneurons and some other SN^26^.

As mRNA samples were extracted from floating spheroids, this could suggest that although neurons can express these markers at the beginning on their own, in a long term, neurons are not able to sustain the expression of proteins destined to form functional connections in the absence of the target tissue (e.g. the muscle). This could be a way of saving resources from neurons and, it could also be linked to the effect of cytokines and growth factors secreted in native conditions by innervated cells. Similar phenomena have been reported to occur in the neuromuscular junction formation, where the post-synaptic domain is first formed in the muscle and together with Schwann cells, they send signals to the motoneuron axon to form the synapse^63,64^. Results suggest that the expression of ETV1 and VGluT1 required to form functional connections, could be induced by cytokines and growth factors secreted by target cells. This highlights the relevance of a biomimicking environment to induce an adequate genetic expression. On top, ROCK inhibitor Y27632 induced in healthy cells a significant decrease of myelin associated protein (MBP +) probably related to the decrease of peripheral neurons (PRPH), a small decrease of GFAP and a big decrease of MN marker (MNX1 +) (Fig. 4 and Fig. S2, *protocol D* vs *protocol A’*). The effect of ROCK inhibitor Y27632 onto ALS cells could be observed in the same genes, in an opposite manner: the expression of PRPH, MBP, GFAP and MNX1 was incremented. This suggests that ROCK inhibitor Y27632 could interfere not only with hNSC cell differentiation, but also with ALS genetic pathway.

When comparing ALS cells to healthy cells without ROCK inhibitor (Fig. 4C and Fig. S1, *protocol A’*), we can see that basal ALS cells expression of motor system cells (PVALB +) is lower. Parvalbumin, although frequently used as a proprioceptive sensory neuron marker, is also expressed in other cells of the motor system, including but not limited to motoneurons and interneurons within the spinal cord and muscles^59,65,66^. ALS cells show also a lower genetic expression of ETV1, myelin basic protein (MBP), and motoneurons (MNX1 +). ETV1 expression is linked to NT3-TrkC signalling on pSN^60,66^, but it is also critical in the formation of functional connections between pSN and MN^66^. This may suggest that despite following the same protocol, ALS cells form a smaller population of myelinated MN that fails to form successful connections with pSN, as observed by significantly lower levels of ETV1, even lower than healthy cells at the initial timepoint. ETV1 could hypothetically interfere with the motor feedback from pSN. Studying the connection between pSN and MN in ALS cells would help elucidate the role of ETV1 and determine if it could be a feasible treatment target.

Furthermore, the expression of certain genes assessed related to the pSN genetic profile is highly upregulated for ALS samples for NTRK3 and DICER, similar for RUNX3 and downregulated for ETV1 and PVALB (Fig. 4E). RUNX3 is known to have a different regulation in each subtype of NTRK3 + neuron^67^. Taken together, these results suggest that pSN related gene basal levels are different and regulated differently in ALS cells compared to healthy cells. And the cell state interferes not only with the differentiation of hNSC, as expected, but also on the genetic profile in different way in healthy and ALS cells.

Considering the differentiation of hNSC to MN spheroids, previous results reported in the literature^33,68^, showed through imaging techniques the expression of stemness and motoneuron differentiation markers (Nestin, Olig2, Sox1, Tuj1, MAP2) in 2D cultures^33^, and neural differentiation markers (HB9, Tuj1, islet1, ChAT, F-actin) in spheroid form^68^. There is previous publications comparing SN and MN organoids obtained from rodent tissue preparations^69^, however to our knowledge there are no previous studies comparing SN and MN obtained from a human cell source. Using MN differentiation as a reference, we took advantage of the protocol established to obtain SN spheroids to compare MN and SN spheroids differentiated from the same hNSC cell source. From the morphological and immunostaining analysis of SN spheroids growth during their differentiation and compared to the one of motoneurons obtained from the same cell source we observed a four-fold increase in the projected transversal area of floating MN spheroids, and an increment of the cells in the corona of plated MN spheroids, lacking in SN spheroids (Fig. S6 and Fig. S7). This suggests that neural proliferation ends up before plating the spheroids, with the differentiation, but the proliferation of cells located outside the core, presumably glial cells although it should be further assessed, continues in MN spheroids. Furthermore, SN spheroids seeded together present a migration trend ending up with the fusion of two nearby spheroids (Fig. 3A). The immunostaining confirmed lower proliferation on cells outside the core of SN spheroids, their fusion of spheroids and lower differentiation specificity (Fig. 3), compared to MN as observed in Fig. 5.

The compartmentalised coculture in Xona microfluidic devices (Fig. 2) showed that SN spheroids were able to extend neurites within the SN compartment and cross to the SkM compartment (Fig. 6). However, as expected from the results obtained in the qPCR (Fig. 4B), the differentiation protocol utilised generated a diverse population of SN, guaranteeing a small percentage of TrkC + sensory neurons. Regarding SkM cells, results suggest that muscle fibres were not completely differentiated. They formed a mix between aligned structures (Fig. S8), and similar morphologies to bag fibres (Fig. 6D), with a cluster of non-aligned nuclei located in the equatorial side of the fibre, comparable with similar ones obtained before in vitro with human samples^47^. However, α-actinin, phalloidin and MHC staining did not show in the SkM a uniform striated distribution of myofibers in the sarcomere (Fig. 6D and Fig. S8). The induction of differentiation of myoblasts into myocytes can be affected not only by the components of the medium. The initial cell confluency and ability to proliferate determines the formation of multinuclear muscle fibres, as cells need to fuse. And in the case of a coculture, the differentiation could also be influenced by the lack of electrical, optical or mechanical stimuli. Furthermore, the differentiation of SkM and SN neurite extension could be influenced by paracrine signalling between them, and therefore, also by the coculture window of time, as reported for MN in a parallel work performed in collaboration^70^. In our study, cells were cocultured at an intermediate timepoint (after 10 days of proliferation and differentiation for SkM cells, and 10 days of differentiation for neural spheroids, before inducing their final differentiation to SN). Previous publications have shown that placing together fully differentiated SkM cells (after 14 days of culture) with fully differentiated MN spheroids for a coculture period of 14 days, produce functional interactions^71^. However, previous studies coculturing SN and SkM have suggested that it is preferable to induce partial differentiation before placing both cells together, as certain factors released by SN (such as neuregulin) could play a role in initiating downstream differentiation of intrafusal muscle fibres and spindles^43^.

There is little literature on in vitro coculture of sensory neurons with skeletal muscle cells^43,48–50^, and no publications seeding them in compartmentalised microfluidic culture systems, The interaction between SN and SkM cells in in vitro cultures has only been analysed through imaging techniques in a few publications^43,48,50^. Rumsey et al*.* have reported the formation of ASW and FSE using rat primary neurons and rat primary myocytes with chain fibre or nuclear fibre morphologies, respectively^48^. Guo et al*.* observed FSE and ASW formation between human neural progenitors derived to sensory neurons and human skeletal muscle stem cells derived to intrafusal fibres^43^. And Qiao et al*.* have published immunostaining images of the interaction of rat primary DRG neurons (in some cases TrkC positive) with rat primary skeletal muscle cells^50^. So far, results obtained here represent a first approach on the interaction between TrkC + neurons and skeletal muscle cells, both from human origin, cultured in vitro using compartmentalised microfluidic devices.

In the microfluidic devices, some of the axons that reached a muscle fibre in the SkM compartment (Fig. 6) were TUJ1 + and TrkC +. Some studies have previously reported finding low levels of TrkC in human muscle^72–74^. The distribution of TrkC within non neural tissues in humans is not well studied. The presence of neurotrophin receptors is known to be related to the differentiation and innervation of the muscle tissue on neural tissues, proprioceptive neuron survival and development of the muscle spindle^75^. However, there are controversies about its presence on muscular tissue. Neurotrophin receptors are expressed mostly during developmental stages of myoblast before they fuse^75^. Some authors report finding significant levels of TrkC in skeletal muscle from developing chicken embryos^76,77^, but not from mice embryos^76^ or on cell cultures from newborn rat tissue^50^. These variable findings could indicate a different expression among species, and/or be related to regeneration or maturation of the muscle, as found for p75NTR in skeletal muscle tissues ^78^. In Fig. 6E, the axon at the top (TrkC +), wraps the SkM fibre with a morphology that suggests the formation of an ASW. The join immunostaining of Tuj1 and TrkC + observed in the image is compatible with synaptic bouton-like structures and this effect was not observed in TrkC- axons. This phenomenon can be better distinguished in 3D reconstruction images (Fig. 7, Fig. S9, Movie S1, Movie S2) and orthogonal views (Fig. 6G–I). Similar structures have been imaged by Qiao et al*.*^50^ in SN terminals of rat primary DRG neurons connecting with intrafusal bag fibres, without assessing the type of SN or recreating the 3D structure. There is a controversy about this topic, as it has been reported that human primary terminals do not have ASW structure^17^. However, the in vitro studies performed by Guo et al.^43^ coculturing sensory neurons and intrafusal muscle fibres from human origin, they observed that sensory terminals form structures with a similar morphology to ASW.

Using the microfluidic device, results showed a limited efficiency on the targeted differentiation, that was still good enough to generate few limited interactions with SkM fibres. The SkM-pSN interaction efficiency could probably be encouraged targeting the differentiation of SkM towards intrafusal muscle fibres^47^. Nevertheless, we detected the formation of synaptic bouton like structures in the contact points of an annulospiral wrapping in the muscle spindle structure. Further analysis should also include the interaction between ALS pSN and ALS model of SkM intrafusal muscle fibres.

The characterisation of cells differentiated to SN showed significant differences for some genes for healthy and ALS samples. Assessing and understanding the pSN subtypes present in healthy and ALS cells would help elucidating their role in the disease. A further analysis of SN genome sequencing, crosschecked with patient databases of genes known to be altered in ALS, should throw some light.

Moreover, improvements of the process, including the incorporation of α-MN into the microfluidic model could help modulating and isolating the effect of SN, as compared to MN, in a more physiological environment with functional stimulation, being able to assess to what length muscle spindle afferents are responsible for triggering MN degeneration in ALS^27,55^, and to evaluate their similarities in the mechanisms involved in the failure, maintenance and repair occurring in ALS^27^. And finally, the addition of γ-MN to recreate the fusimotor system calibrating the sensitivity of the spindle, could help understanding its resistance mechanisms to neurodegeneration in ALS^13,52,53^, and to understand to what length is stretch-sensitivity feedback of the muscle spindle related to the development and progression of ALS^13,54^.

To sum up, the protocol established to obtain proprioceptive sensory neurons from healthy and ALS neural stem cells has enabled the obtention of a set of pSN subtypes biomimicking a natural environment as cells provide their native support to each other. Moreover, TrkC + cell population is known to be dynamic throughout the differentiation process^37^, and pSN subtypes have some variations in their genetic profile, such as RUNX3^67^. The experiments showed that ROCK inhibitor Y27632 (a medium component in *protocols C* and *D*) could interfere not only with neural stem cell differentiation, but also with the ALS genetic pathway. Besides, ALS samples showed altered pSN related gene expression and regulation, together with lower ETV1 levels (required for a functional connection between pSN and motoneurons). This suggests that pSN alteration could potentially be involved in ALS pathology.

## Methods

### Experimental design

The objectives of this study were to optimise a protocol for human pSN differentiation, to recreate pSN connection with SkM on a chip, and to evaluate the role of pSN in ALS development. After establishing a protocol to differentiate human neural stem cells into sensory neurons, a robust model of SN-SkM connection was created in vitro in compartmentalised microfluidic devices. A diagram of the differentiation and coculture timepoints is shown in Fig. 2. The composition of all culture media used is described in Table S3. All samples containing living cells were maintained at all times in a CO_2_ incubator at 37 °C and 95% humidity. The use of commercial cell lines of human iPSC-derived NSCs was approved by the respective departments at Massachusetts Institute of Technology. All techniques performed with human cells were performed in accordance with National Academy of Sciences Guidelines for Human Embryonic Stem Cell Research and with the Massachusetts Institute of Technology Committee on Assessment of Biohazards and Embryonic Stem Cell Research Oversight (CAB/ESCRO).

### Culture of hNSC and differentiation of pSN

The cells utilised were healthy human neural stem cells (hNSC) H9-derived (a commercial neural stem cell cell-line from Gibco, obtained from a healthy female donor, #N7800100) and human sporadic ALS iPS-derived neural stem cells (a commercial neural stem cell cell-line from iXCells Biotechnologies obtained from a 55 years old Caucasian female sporadic ALS donor without any known mutations in SOD1 or C9ORF72, #40HU-007). Both healthy and ALS hNSC were seeded onto Matrigel-coated surfaces prepared by making 1:30 dilution of Matrigel^®^ hESC-Qualified Matrix (Corning, #354277) into Knockout™ DMEM/F-12 medium, also known as Dulbecco's Modified Eagle Medium/Nutrient Mixture F-12 (Gibco, #12660012) incubated for 1 h at room temperature. Cells were maintained with *hNSC proliferation medium* changing it every day after. Once the desired number of cells was reached, at TP0, cells were washed with sterile DPBS, also known as Dulbecco’s Phosphate Buffered Saline (Lonza, #17-512F), detached with TrypLE™ express enzyme (Gibco, #12604021), counted with a haemocytometer and transferred in the desired density to ultra-low attachment PrimeSurface^®^ 3D culture spheroid plates (S-Bio, #MS-9096MZ) to form neurospheroids.

### Differentiation of pSN from hNSC

To optimise the differentiation protocol, five different conditions were assessed (*protocols A-D*) summarised in Table S4. In the case of *protocol A and A’*, we performed each at least 3 times. For the other ones, once we assessed the qPCR result, we discarded and discontinued repeating them for other different characterization purposes (e.g. immunostainings). The initial medium assessed (*protocol A*) was adapted from a previous publication^35^. The increase of NT-3 concentration and addition of CHIR99021 and ROCK inhibitor Y27632 at different steps of the differentiation were assessed adapting the process (*protocols A’-D*). These three components and their combinations were chosen based on their biological relevance on neurogenesis and differentiation towards pSN. Neurotrophin-3 (NT-3) is expressed in muscles and limbs, implicated in target recognition and survival for MN^79^, and known to play an important role in proprioceptive development^80^. ROCK inhibitor Y27632, is known to promote neuronal differentiation of iPSC, neurogenesis and migration of neural stem cells, neurite outgrowth of sensory neurons in vitro, and enhance the regeneration of motor axons^81^. Low doses are known to mediate pro-nociceptive responses, whereas high doses reduce edemas^81^. CHIR99021, also known as glycogen synthase kinase 3 (GSK3) inhibitor, is a Wnt signalling pathway agonist. It promotes extracellular matrix remodelling by inducing the expression of matrix metalloproteinases^82^, it can promote proliferation of neural progenitors^83^, and axon regeneration in sensory neurons^84^. It also facilitates the differentiation towards neural crest cells, being reported by higher levels of p75 neurotrophin receptor (p75NTR) and whilst maintaining similar levels of HNK-1 to other protocols^44^. Furthermore, a specific type of ALS characterised by mutations in the spastic paraplegia gene 11, is known to have GSK3 pathway dysregulated in neural progenitors, causing as a result motor neuron degeneration and peripheral sensorimotor neuropathies^85^. Modulating GSK3, it is possible to rescue mitotic activity of neural progenitors^85^.

hNSC seeded at TP0 were kept with *hNSC medium* for 8 days (until TP1). During the 10 following days, to promote the beginning of the differentiation towards neural crest stem cells, the medium was progressively changed from *medium hSN2A* to *medium hSN2B*, changing its composition every two days. In other words, at TP1 cells were kept for two days with 100% *medium hSN2A*, then two days with 75% *medium hSN2A* and 25% *medium hSN2B*, then two days with 50% of each media, then two days with 25% *medium hSN2A* and 75% *medium hSN2B*, remaining finally for two extra days with 100% *medium hSN2B*. During this progressive differentiation, in some experimental conditions CHIR99021 was freshly added on every change of medium. After the last two days with 100% *medium hSN2B*, at TP2, cells were changed to *medium hSN3*. During this step, in some experimental conditions ROCK inhibitor Y27632 was freshly added on every change of medium. This medium was kept until the endpoint of the experiment, for 22 days, changing it every day after.

### Characterisation of differentiated pSN

hNSC samples undergoing pSN differentiation protocols were analysed at intermediate (TP2) timepoint to assess neural crest stem cells markers and at final timepoint (TP3) through qPCR and immunostaining evaluating the expression of several genes (summarised in Table S2).

#### Flow cytometry analysis during pSN differentiation at intermediate timepoint

At TP2, samples of healthy and ALS hNSC undergoing *SN differentiation protocol A* differentiated as 2D cultures ($57.000 {cells\cdot cm }^{-2}$) and spheroids of different seeding densities (${\uprho }_{1}= 3.000 cell\cdot {spheroid}^{-1}$; ${\uprho }_{2}= 4.500 cell\cdot {spheroid}^{-1}$; ${\uprho }_{3}= 6.000 cell\cdot {spheroid}^{-1}$) were analysed to understand the influence of the seeding density and the spheroid on the initial differentiation, within the working range. A further analysis was performed onto healthy and ALS hNSC undergoing each of the other differentiation protocols (A’-D) in the form of spheroids (${\uprho }_{3}= 6.000 cell\cdot {spheroid}^{-1}$) to assess the effects of each differentiation protocol onto the initial steps of the differentiation. In all cases, the analysis was performed looking for HNK1 + cells, a neural crest stem cells marker^46,51^.

To that end, cells were washed with DPBS, and trypsinised incubating 5 min at 37 °C with TrypLE (Gibco, #12604021) in the case of 2D cultures or Accumax (Innovative Cell Technologies, #AM105) for spheroids. Then samples were placed in a washing buffer made of 5% foetal bovine serum (FBS) in DPBS. From this moment samples were kept in ice all the time. Cell suspensions were filtered and placed into flow cytometry tubes with 75 µm filters (Falcon, #352235). Then they were centrifuged for 4 min at 200 *g* and after aspirating the supernatant, the pellet was resuspended in 100 µL of washing buffer. The staining was performed incubating samples for 15 min with a solution of 1:200 dilution of HNK1-FITC (Biolegend, #359603) in washing buffer. Then samples were diluted 5 times in washing buffer to increase the sample volume and analysed in a BD™ LSR II flow cytometer. The data obtained were processed using Flowing Software version 2.5.1^86^. Further visualization and statical analysis were performed with Python custom script using FlowKit package (version: 0.9.1). Gating strategies for identifying HNK1 positive cells were shown in Fig. S4. The gating thresholds in Fig. S4 in HNK1-FITC were determined according to 95% confidential interval of negative non-staining control.

#### Imaging of floating spheroids during pSN differentiation at intermediate timepoint

At TP2, spheroids were washed with DPBS and then fixed and permeabilised with Cytofix/Cytoperm (BD Biosciences, #51-2090KZ) for 30 min. After a 3 min centrifugation at 1000 rpm, samples were washed in tubes with DPBS. Spheroids were immobilised by embedding them in a collagen neutralised solution and placing them in glass bottom multiwell plates. The composite solution was prepared mixing 8 parts of collagen cell matrix type I-A (Wako Chemical, #631-00651), 1 part of ${NaHCO}_{3}$ 0.05 N (Sigma Aldrich, #S5761), and 1 part of balanced and concentrated Ham-F12 10X medium (Wako Chemical, #630-29661), as previously described^71^. The collagen with spheroids was incubated for 10 min at 37 °C to enable the polymerisation.

For a later imaging, the immunostaining procedure was performed as follows, with all steps made at room temperature unless stated otherwise. Floating spheroids were washed three times with DPBS for 10 min, permeabilised with DPBS 0.1% triton (Sigma, #T9284) for 10 min, and incubated for 2 h with a blocking solution containing 1% bovine serum albumin (BSA, Sigma, #A9657) in DPBS. Samples were then washed 3 times with DPBS for 10 min, and incubated overnight at 4 °C with the primary antibody solution containing 0.1% triton, 1% BSA, 0.01% sodium azide (Sigma, #71289), and 1:500 dilution of mouse anti-TUJ1 antibody, also known as anti-beta III Tubulin (Abcam, #ab78078) in DPBS. The day after, primary antibody solution was removed, and samples were washed 3 times with DPBS for 10 min. Then samples were incubated for 2 h protected from light with a secondary antibody solution containing 0.1% triton, 1% BSA, 0.01% sodium azide, and 1:150 dilution of HNK1-FITC (Biolegend, #359603) in DPBS. From this moment, samples were kept protected from light throughout the whole procedure. After secondary antibody solution was removed, samples were washed three times with DPBS for 10 min. Then samples were incubated for 10 min with 14.3 µM of DAPI (4', 6-Diamidino-2-Phenylindole dihydrochloride, Invitrogen™, #D1306) in DPBS. Samples were washed with DPBS and mounted with Olympus scaleview solution (Olympus, #ScalView-A2 optics). Imaging was performed with a confocal laser scanning microscope (Olympus, #FV1200) and images were processed afterwards with ImageJ software^87^.

#### Real time PCR (qPCR) for mRNA analysis

The mRNA of healthy SN spheroids following differentiation *protocol A* was quantified to assess the relative evolution in gene expression. The mRNA of healthy and ALS spheroids following each differentiation *protocol (A’-D)* was quantified at the final timepoint to find differences in gene expression in healthy and ALS samples and to assess the effect of each differentiation protocol. For that purpose, after doing cell lysis, RNA was isolated, converted to cDNA and then real time polymerase chain reaction or quantitative PCR (qPCR) was performed.

For each sample two spheroids were washed with sterile DPBS and incubated with 100 µL of Accumax (Innovative cell technologies, #AM105) for 5 min at 37 °C. The cell suspension of two spheroids was mixed together, neutralised in 5 mL of DMEM supplemented with 10% FBS, and centrifuged for 4 min at 3.5 *g*. After aspirating the supernatant, samples were placed on ice. The mRNA was isolated using the manufacturer’s protocol of RNeasy mini kit for RNA isolation (Qiagen, #74104), RNase free water (Ambion, #AM9938), RNase spray for hands (Ambion, #AM9782), RNase free tubes and micropipette tips and keeping samples during the whole process in ice. Once the mRNA was obtained, reverse transcription was performed to obtain cDNA using SuperScript VILO cDNA Synthesis Kit (Invitrogen, #11754-050) and a thermocycler (Eppendorf, MasterCycler personal). We analysed each sample obtained from two spheroids at least 3 times through qPCR. Afterwards, the obtained cDNA was quantified using a nanodrop (Thermofisher, Nanodrop 200) to check for the purity and concentration of nucleic acids.

Forward and reverse primer sequences of each gene of interest (summarised in Table S5) were chosen according to PrimerBank database^88^, checking for NCBI Gene ID, and purchased from IDT (Integrated DNA Technologies, Inc., U.S.). The cDNA samples were incubated with the mastermix and dye—TB Green^®^ Premix Ex Taq™ II (Clontech Takara, #RR820L)—, RNase free water, and the forward and reverse primers for each gene of interest. Samples were loaded in optical 384 well plates (MicroAmp, #4309849) filling 3 wells for each target gene and sample. Then the plate with three technical replicates was covered with optical adhesive films (MicroAmp, #4,311,971), and centrifuged for 2 min at 2.000 rpm before reading the plates in a 7900HT Fast Real-Time PCR System (Applied Biosystems) performing for the amplification an initial step of 2 min at 50 °C, followed by 50 cycles of 5 s at 95 °C and 30 s at 60 °C, and finishing with a dissociation stage of 15 s at 95 °C, 15 s at 60 °C and 15 s at 95 °C, to assure the specificity of qPCR measurements.

The obtained ${C}_{T}$ values for each sample were analysed following the Livak method for relative analysis of gene expression^89,90^. First, for each sample and gene read (${C}_{T}(sample,gen)$) the average of the four technical replicates was calculated. Then, the three housekeeping readings (ACTB, GAPDH, RPS18) were used as internal standard control to calculate the ${\Delta C}_{T}$ for each sample, gene and housekeeper, as indicated in the following equation:

$${\Delta C}_{T}= {C}_{T}\left(sample,\, gene\right)- {C}_{T}(sample,housekeeper)$$

In this way, for each sample-gen-timepoint combination, we obtained three measurements of ΔCt (against B-ACTIN, GAPDH and RPS18S).

To perform relative analysis of gene expression, the calibrator sample was set as *protocol A* TP1 for the analysis of healthy samples undergoing *SN differentiation protocol A* at different timepoints. Healthy and ALS samples undergoing all protocols at TP3, were analysed twice, stablishing both results from *protocol A* TP3 and results from *protocol A* TP1 as calibrators. Testing samples were compared against calibrator samples:

$${\Delta \Delta C}_{T}= {\Delta C}_{T}\left(test\right)- {\Delta C}_{T}(calibrator)$$

Fold change was calculated as follows for each gene against each housekeeper:

$$Fold \, change = {2}^{-\Delta \Delta {C}_{T}}$$

Then the normalised expression ratio of the expression of each sample and gene against each housekeeper was calculated as follows:

$$Normalised\, expression\, ratio = {log}_{2}({2}^{-\Delta \Delta {C}_{T}})$$

Finally, the mean fold change was calculated performing the average of the normalised expression ratio for each gene and sample against each of the three housekeepers. The average of normalised mean fold change was plotted represented in a colour-code matrix (Fig. 4A).

$$Normalised\, mean \,fold \,change\,=\,average\, ({Normalised \,expression\, ratio}_{housekeeper})$$

The average of ΔCt values (normalised against the three housekeepers for each readout), was also calculated and plotted in Fig. S1 and Fig. S2. Raw Ct values of housekeeper genes were calculated and plotted in Fig. S3.

Statistical analysis was conducted with GraphPad Prism program. D’Agostino-Pearson normality test was conducted in all sample datasets. To test the increase or decrease of target gene expression in time, ΔCt results (normalised against the housekeeper) were plotted in a graph and, for each gene and protocol, Mann–Whitney test against TP1 was conducted (Fig. S1). To test the differences among protocols for each gene, data from TP3 were analysed through non-parametric Kruskal–Wallis test followed by Dunn’s post-hoc test (Fig. S2). To test the stability of housekeepers between healthy and ALS samples, Mann–Whitney statistics test was conducted for unpaired samples (Fig. S3). Differences between experimental groups were considered statistically significant at p < 0.05.

#### Imaging of plated spheroids at TP3

In order to check the differentiation of healthy spheroids, an immunostaining was performed at TP3 as described below in the inmunostaining section.

### Comparison between SN and MN

#### Differentiation of hNSC to motoneurons

The differentiation from hNSC to motoneurons was conducted following a previously published protocol^33,68^. Briefly, hNSC seeded at TP0 were kept with *hNSC medium* for 8 days (until TP1). At TP1, the medium was replaced with *hMN medium* (see composition in Table S3) to induce motoneuron differentiation and maturation. This medium was maintained until the endpoint of the experiment at TP3 (i.e. 30 days for comparison with SN), changing it every day after.

#### Comparison between hNSC differentiated to SN and MN

Several morphological features of spheroids undergoing *MN differentiation protocol* and *SN differentiation protocol A* were assessed and compared with each other as floating and platted spheroids.

For the floating spheroid analysis, hNSC spheroids of same cell densities (${\uprho }_{1}= 3.000 cell\cdot {spheroid}^{-1}$; ${\uprho }_{2}= 4.500 cell\cdot {spheroid}^{-1}$; ${\uprho }_{3}= 6.000 cell\cdot {spheroid}^{-1}$) were cultured as individual floating spheroids following *MN differentiation protocol* or *SN differentiation protocol A* and analysed at different timepoints. Bright field images were taken from 15 ddiff every 3–4 days until TP3 using an inverted microscope for transmitted light (Zeiss, #Axiovert-200). The maximum transversal area of spheroids was quantified with ImageJ software. Mean and standard deviation of obtained result was quantified for n = 3 samples. Results obtained for MN and SN spheroids were compared and statistical analysis was conducted with GraphPad Prism program. Experimental data were analysed with D’Agostino-Pearson normality test. Data from each timepoint measurement could not be taken as normally distributed due to a low sample number, and were processed through non-parametric Mann–Whitney statistics test for unpaired samples, resulting in non-significant differences due to a low sample number (n = 3). Data from grouping all samples of MN versus all samples of SN, normally distributed, were anaysed by parametric t-test for unpaired samples, followed by Welch’s correction for samples without assuming equal SDs. Differences between experimental groups were considered statistically significant at p < 0.05.

For plated spheroids, hNSC spheroids of an initial cell density of $6.000 cell\cdot {spheroid}^{-1}$ were cultured, following *MN differentiation protocol* or *SN differentiation protocol A,* and transferring them to Matrigel coated plates at TP2. To perform the plating, after aspirating the Matrigel solution, one or two SN or MN spheroids with up to 300 µL of the required medium were placed in the middle of each coated well, handling them with wide orifice tips (VWR, #736–0205). Samples were incubated for approximately 4 h to let spheroids adhere to the bottom of the plate. Then additional 200 µL of medium were gently added. Spheroids were maintained in a ${CO}_{2}$ incubator at 37 °C and the medium was changed every day after until TP3. Bright field images were taken every 3–4 days, at the same timepoints for MN and SN spheroids, using an inverted microscope (Zeiss, #Axiovert-200). Images were analysed using ImageJ software. For each image taken (n = 2), the core area (considered as the area occupied by the central circular shape of the neurospheroid), the corona area (considered as the area occupied by the surrounding cells and neural projections) and the spheroid approximation (considered as the inter-spheroid distance shortening caused by spheroid migration) were observed.

At the final timepoint, plated spheroids were fixed incubating them with a 4% paraformaldehyde solution for 30 min at room temperature. Then samples were washed three times with DPBS and stored at 4 °C until the immunostaining was performed.

### Coculture of SN and SkM cells

#### Culture and differentiation of SkM

Healthy iCell® skeletal myoblasts (Cellular Dynamics International—Fujifilm, U.S.) were seeded onto Matrigel-coated surfaces. Human skeletal myoblasts (hSkMb) were maintained with *hSkMb proliferation medium* changing it every day after. Cells were kept proliferating for 6–10 days before reaching subconfluency. At that point the *SkM differentiation protocol* was started*,* adapted from a previous publication^68,71,91^. Briefly, to induce differentiation to human skeletal myocytes (hSkMc), once hSkMb had reached subconfluency, cells were incubated with *hSkMc differentiation medium*, increasing progressively horse serum percentage from 2% during 4 days in vitro (DIV), to 4% for another 4 DIV, and finally up to 10% to the endpoint of the experiment at TP3. The medium was always changed every day after.

Bright field images were taken at different timepoints to monitor proliferation and differentiation. For a later imaging, at TP3 cells were fixed in 4% paraformaldehyde and stained following the immunostaining procedure described later.

#### Preparation of Xona microfluidic devices

For the compartmentalised coculture in 2D, Xona microfluidics commercial devices with microchannels of 900 μm (length), 10 µm (width), 5 µm (height) and spaced 50 µm apart (Xonamicrofluidics, #SND900, US) were utilised.

To prepare the devices, a 2 mm perforation was made in the SN seeding side with a punch (Miltex Instruments, #33-31, US), and a 3 mm perforation in the SkM seeding side with another punch (Miltex Instruments, #33-32, US), as shown in Fig. 2B for chambers c1 and c2, respectively. Then, the glasses (Fisher scientific, #12-542-C, US) and the Xona polydimethylsiloxane (PDMS) devices were cleaned sonicating in 100% ethanol for 15 min, drying with a nitrogen gas stream and dehydrating in a drying oven at 60 °C for at least 30 min (Binder, #FD-23). Finally, glass coverslips were permanently bonded to the PDMS pieces exposing both to 1 min under high frequency oxygen plasma (Harrick plasma, #PDC-001) at 700 mTorr, then putting both pieces together and sealing them with at least 30 min dehydration in the oven at 60 °C. At this point, devices could be stored for the beginning of the experiment.

#### Device assembly and cell culture in Xona devices

To perform the cell culture of SN spheroids following *differentiation protocol A* with hSkMc, previously prepared devices were activated, sterilised, and coated before seeding cells in them. First the glass and PDMS surfaces were activated applying 5 min of medium frequency oxygen plasma, then adding sterile de-ionised water. At this point devices were transferred to the hood, where they were sterilised through 15 min of UV radiation and rinsed with sterile water.

After aspirating all the water, the coating was performed from the inlet and outlet reservoirs (indicated in Fig. 2B as R1i, R1o, R2i, R2o), adding 200 µL of Matrigel^®^ hESC-Qualified Matrix 1:30 in Knockout™ DMEM/F-12 medium in R1i and R2i, and after letting volumes equilibrate, adding extra 150 µL in R1o and R2o, to avoid in this way microbubbles inside the channels. The coating was performed by incubating devices for 1 h at room temperature.

Once devices were coated, the coating solution was aspirated and they were filled with *hSkMb medium* in the same way: putting 200 µL on R1i and R2i, letting volumes balance, and putting 200 µL on R1o and R2o. Then, previously trypsinised SkM cells were counted and, for each device, approximately 40.000 cells were resuspended in 10 µL of *hSkMb medium* and injected slowly in the seeding port (the perforated 3 mm hole indicated as *2* in the right side of Fig. 2B). SkM cells were maintained proliferating, changing the medium every day after until TP2. The medium was always changed aspirating the old one and adding the new one from the reservoirs to diminish as much as possible the shear stress onto cells.

At TP2 SkM cells were changed to *hSkMc medium* supplemented with 2% horse serum, changing it on only on the SkM side reservoirs (R2i and R2o). The SN side of the device was washed with DPBS before adding *hSN3 medium* (200 µL on R1i and, after volumes were balanced, 200 µL on R1o). Then, for each device, one SN spheroid was gently handled and inserted in the SN seeding port (the perforated 2 mm hole indicated as *1* in the left side of Fig. 2B), letting the spheroid precipitate to the bottom. The medium for each compartment was changed every day after. SN were kept with *hSN3 medium* until the endpoint of the experiment. SkM followed the differentiation protocol described before.

At TP3 devices were fixed with a 4% paraformaldehyde solution for 30 min and followed the immunostaining procedure described below.

### Immunostaining

All samples observed were maintained in culture until TP3, when samples were fixed incubating them with a 4% paraformaldehyde solution for 30 min at room temperature. Then samples were washed three times with DPBS and stored at 4 °C until the immunostaining was performed.

Samples were first washed with DPBS and permeabilised incubating with DPBS 0.1% triton for 10 min. Then they were incubated with a blocking solution containing 1% bovine serum albumin (BSA, Sigma, #A9657) in DPBS for 2 h. Later they were washed three times with DPBS, incubating 10 min per wash, and incubated with the primary antibody solution overnight at 4 °C. The primary and secondary antibody solution for all cases was a DPBS solution with 0.1% triton, 1% BSA, 0.01% sodium azide and varying antibodies for each cell staining required. For SkM samples, the primary antibody solution contained 1:100 dilution of rabbit anti-MHC antibody, also known as anti-slow skeletal myosin heavy chain antibody (Abcam, #ab173366), and 1:200 dilution of mouse anti-sarcomeric α-actinin antibody (Abcam, #ab9465). MN spheroids, the primary antibody solution contained within 1:200 dilution of rabbit anti-ChAT antibody, also known as anti-choline acetyltransferase antibody (Abcam, #ab181023), and 1:500 dilution of mouse anti-TUJ1 antibody (Abcam, #ab78078). And for SN spheroids, the primary antibody solution utilised included 1:500 dilution of mouse anti-Tuj1 antibody (Abcam, #ab78078), 1:100 dilution of rabbit anti-Pou4f1 (Invitrogen, #PA5-41509) and $1\upmu g\cdot {mL}^{-1}$ of goat anti-TrkC antibody (R&D Systems, #AF373).

After the incubation with the primary antibody solution overnight samples were washed three times with DPBS, incubating 10 min per wash. Then samples were incubated with the secondary antibody solution and from this moment, samples were kept protected from light throughout the whole process. For that purpose, SkM cells were incubated for 2 h at room temperature with a secondary antibody solution that included 1:500 dilution of goat anti-mouse Alexa Fluor 488 (Invitrogen, #A-11029), and 1:500 dilution of goat anti-rabbit Alexa Fluor 555 (Invitrogen, #A-21428). MN spheroids were incubated for 2 h at room temperature with a solution containing 1:500 dilution of goat anti-rabbit Alexa Fluor 488 (Invitrogen, #A-11034), and 1:500 dilution of goat anti-mouse Alexa Fluor 647 (Invitrogen, #A-21235). In the case of SN spheroids, the incubation with the secondary antibody was performed in two steps. SN spheroids were first incubated for 2 h with a solution containing 1:500 dilution of donkey anti-goat Alexa Fluor 633 (Invitrogen, #A21082). Then the solution was removed, and SN samples were washed with DPBS three times, 10 min per incubation. SN spheroids were incubated afterwards for 2 h with a secondary antibody solution including 1:500 dilution of goat anti-mouse Alexa Fluor 488 (Invitrogen, #A11029), and 1:500 dilution of donkey anti-rabbit Alexa Fluor 555 (Invitrogen, #A21428).

After the incubation with the secondary antibody was finished, samples were washed with DPBS three times, 10 min per incubation. Then samples were incubated for 10 min with 14.3 µM of DAPI (4', 6-Diamidino-2-Phenylindole dihydrochloride, Invitrogen™, #D1306) in DPBS, washed with DPBS three times, 10 min per incubation. At this point, some samples were incubated as well with 0.1 µM of rhodamine phalloidin (Cytoskeleton, #PHDR1) in DPBS for 10 min. Then samples were mounted with Olympus scaleview solution (Olympus, #ScalView-A2 optics). Samples were imaged with a confocal laser scanning microscope (Olympus, #FV1200) and images were processed afterwards with ImageJ software. For co-localisation analysis between TrkC and Tuj1 channels, for example, the following calculation was performed:

$$Colocalisation\, percentage = \frac{mean \,gray \,value \,of \,colocalising \,pixels\, (TrkC \,and\, Tuj1)}{mean\, gray\, value\, of\, Tuj1}$$

## Supplementary Information

Supplementary Information.

Supplementary Video 1.

Supplementary Video 2.

## Data Availability

All data used to reach the conclusions in this paper are present in the paper and the Supplementary Materials. Additional data related to this paper may be requested from the authors.
