# Supplementary material for: In vitro modelling of human proprioceptive sensory neurons in the neuromuscular system
Source: Sci Rep. 2022 Dec 9;12:21318. doi: 10.1038/s41598-022-23565-3 (PMC9734133; doi:10.1038/s41598-022-23565-3)
Supplement: Supplementary file 1 — Supplementary Information. [file 41598_2022_23565_MOESM1_ESM.pdf]

Supplementary information

In vitro modelling of human proprioceptive sensory neurons in the neuromuscular system

Maider Badiola-Mateos,[1,2,4,†] Tatsuya Osaki,[4,+] Roger Dale Kamm[4, 5]*, Josep Samitier[1,2,3]*

[1] Institute for Bioengineering of Catalonia (IBEC) - Barcelona Institute of Science and Technology, 08028 Barcelona, Spain.
[2] Department of Electronic and Biomedical Engineering, Universitat de Barcelona, 08028 Barcelona, Spain.
[3] Centro de Investigación Biomédica en Red (CIBER-BBN), 28029 Madrid, Spain.
[4] Department of Biological Engineering, Massachusetts Institute of Technology (MIT), 500 Technology Square, MIT Building, Cambridge, MA, 02139 USA
[†] Current affiliation: The BioRobotics Institute, Department of Excellence in Robotics and AI, Scuola Superiore Sant'Anna, 56127, Pisa, Italy
[+] Current affiliation: Institute of Industrial Science, The University of Tokyo, 4-6-1, Komaba, Meguro-ku, Tokyo, 153-8505, Japan
[5] Department of Mechanical Engineering, Massachusetts Institute of Technology, 500 Technology Square, MIT Building, Cambridge, MA, 02139 USA
* corresponding authors: jsamitier@ibecbarcelona.eu , rdkamm@mit.edu

These supplementary materials include:

- **Figs. S1 to S9**
- **Tables S1 to S5**
- **Movies S1 and S2**

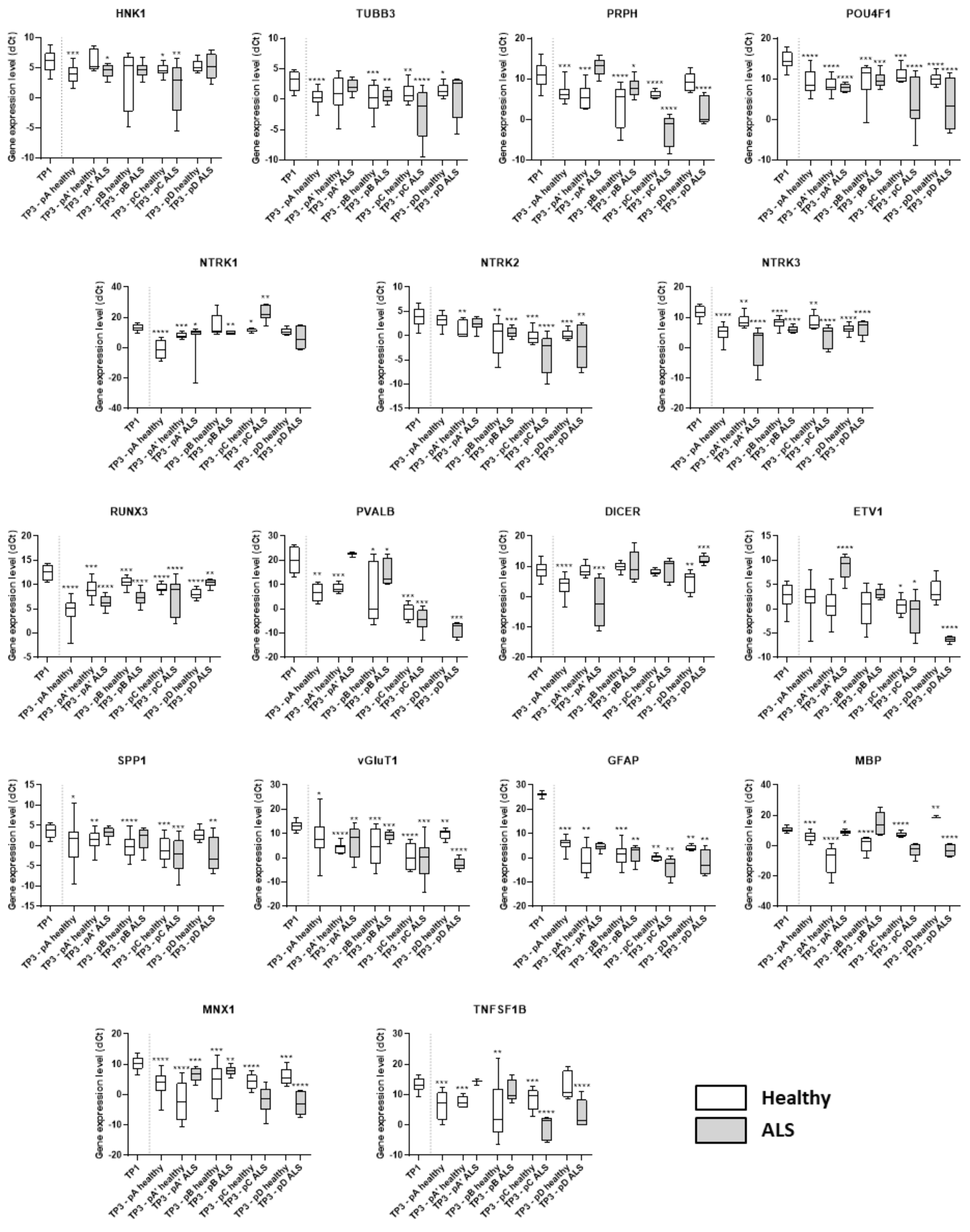

Fig. S1. Gene expression (ΔCt) of target genes at TP1 and TP3. Boxplots with Tukey whiskers represent the gene expression levels obtained in healthy (white) and ALS (grey) samples before any differentiation protocol (at TP1) and with different protocols at TP3. Each value from the graph is obtained normalising Ct raw values against the housekeepers (B-ACTIN, GAPDH, RPS18S) and calculating the average. Higher Ct values indicate later amplification and therefore lower gene expression level. For each gene and protocol, the results of Mann-Whitney test against TP1 results are indicated above each bar with stars. P-values (*<0.05; **<0.01; ***<0.001; ****<0.0001).

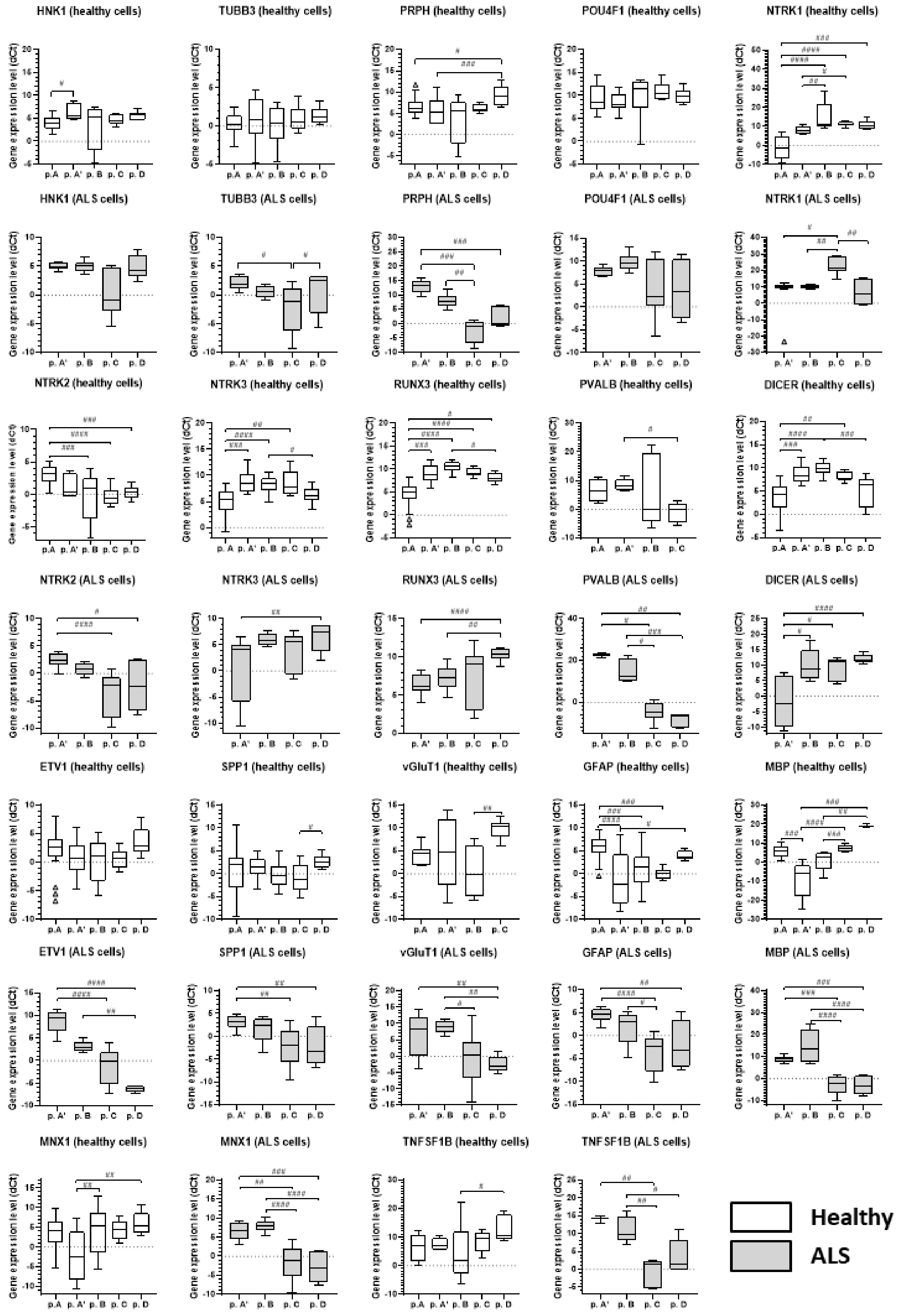

Fig. S2. Gene expression (ΔCt) of target genes at TP3 with different protocols. Boxplots with Tukey whiskers represent the gene expression levels obtained in healthy (white) and ALS (grey) samples with different protocols at TP3. Each value from the graph is obtained normalising Ct raw values against the housekeepers (B-ACTIN, GAPDH, RPS18S) and calculating the average. Outliers are shown with a triangle shape. P-values (#<0.05; ##<0.01;

###<0.001; ####<0.0001) were determined by Kruskal-Wallis test followed by a Dunn's post hoc test. The results Dunn's post hoc test are indicated above the bars with # symbol.

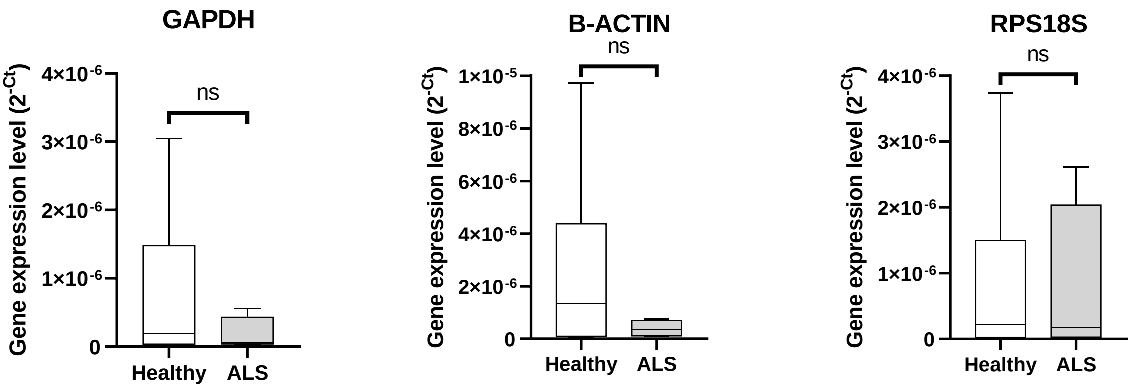

Fig. S3. Gene expression (2^{-Ct}) of housekeeping genes. Boxplots with Tukey whiskers represent the gene expression levels obtained in samples of the gene expression of each housekeeper at TP1 and TP3 with different protocols, clustered in healthy (white) and ALS (grey) samples. Each value from the graph represents obtained raw Ct values. Outliers are shown with a triangle shape. Mann-Whitney statistics test was conducted for unpaired samples, resulting in non-significant differences between ALS and healthy samples.

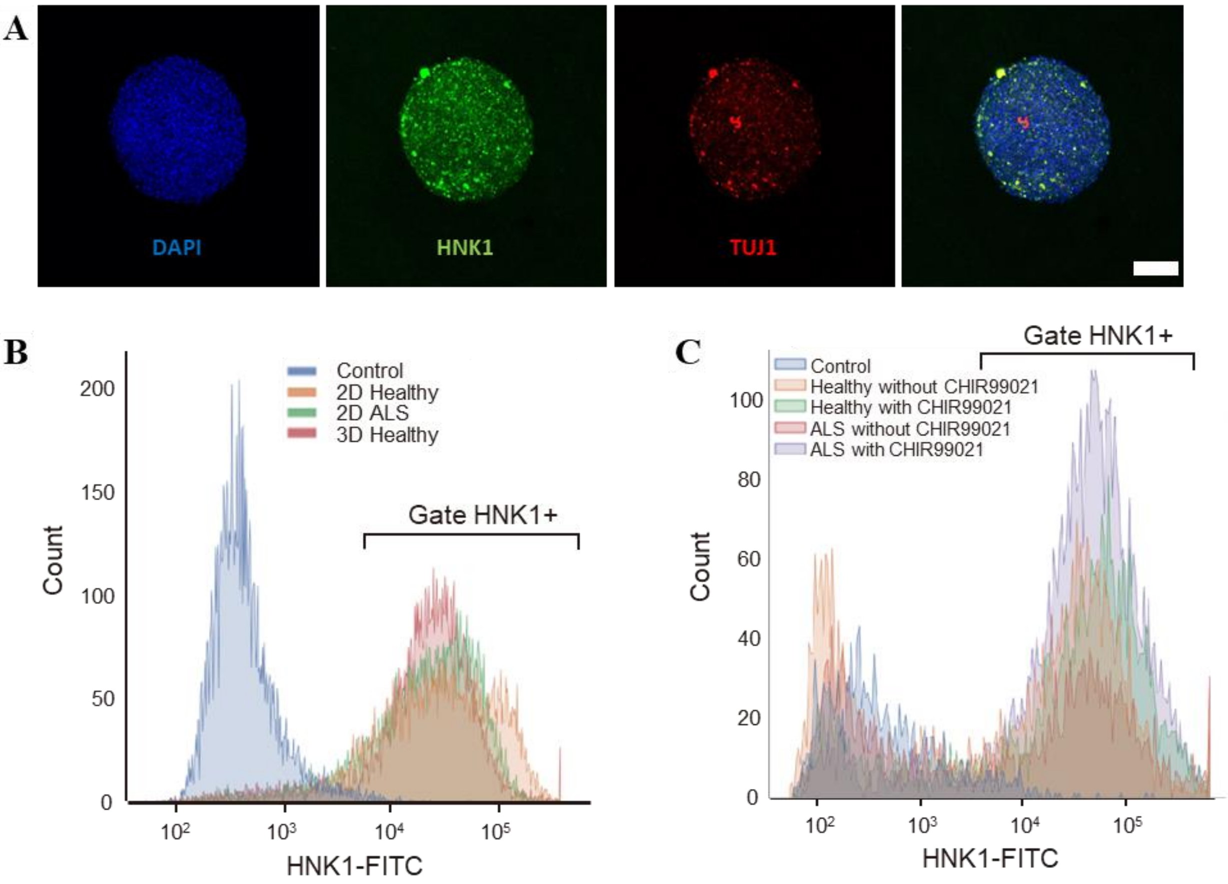

Fig. S4. Characterisation of spheroids undergoing SN differentiation protocols at TP2. A) Floating spheroid immunostaining at TP2. Maximum z-projection of fluorescence images obtained of the immunostaining of healthy samples undergoing SN differentiation protocol-A at TP2. DAPI is shown in blue, HNK1 in green, and TUJ1 in red. Scale bar 100µm. **B)** Flow cytometry analysis of HNK1 on 2D culture of healthy (orange), 2D cultures of ALS (green), and 3D spheroids of healthy samples at different densities (red), all undergoing Protocol A. **C)** Flow cytometry analysis of HNK1 of healthy spheroids undergoing protocols A’ and D (without CHIR99021, in orange), healthy spheroids undergoing protocols B and C (with CHIR99021, in green), ALS spheroids undergoing *protocols A’ and D* (without CHIR99021, in red), and ALS spheroids undergoing *protocols B and C* (with CHIR99021, in purple). In all FC graphs, negative control of non-stained healthy samples is shown in blue.

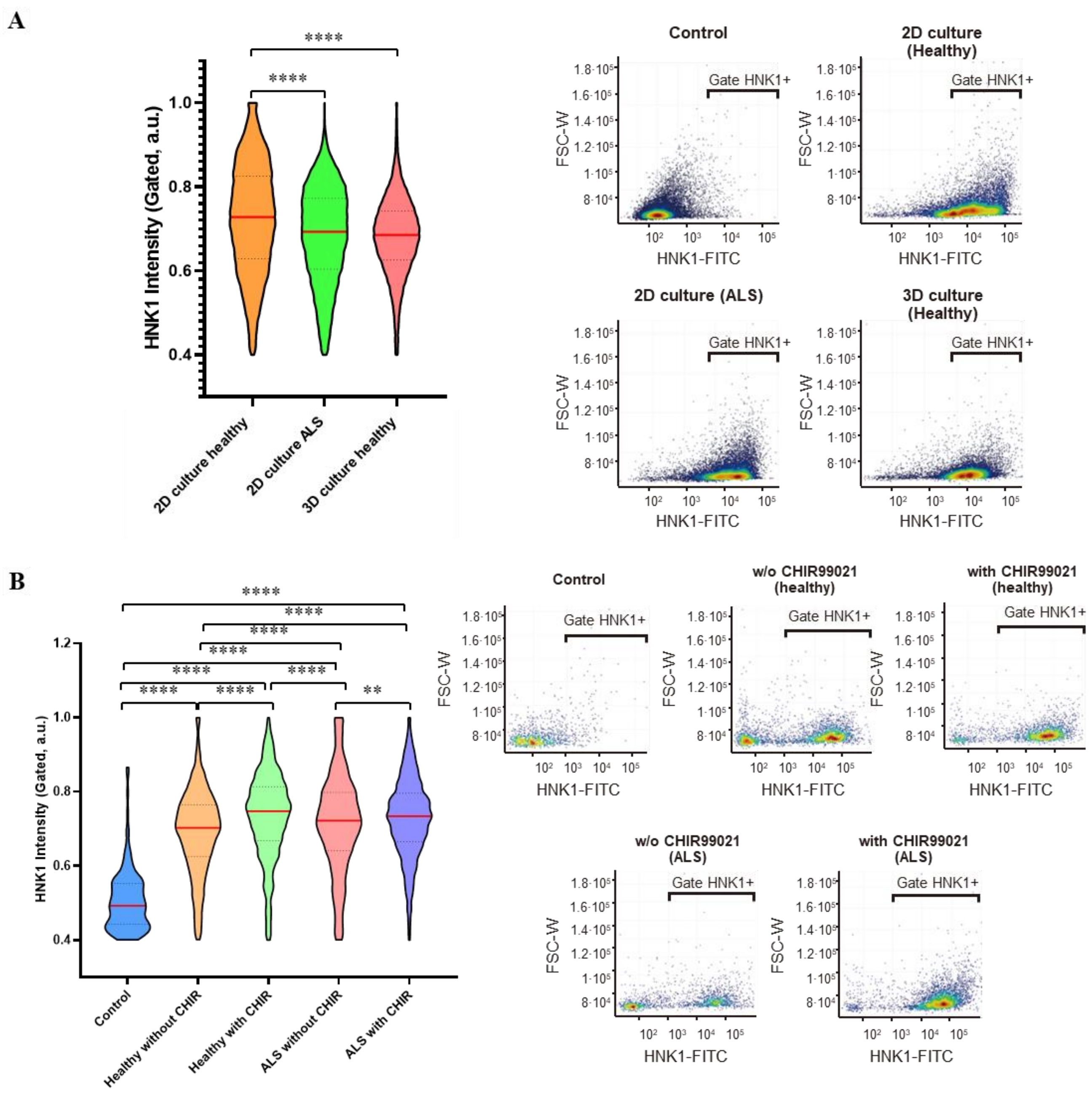

Fig. S5. Scatter plots of flow cytometry analysis, FSC vs HNK1-FIT data, showing the gating strategy for HNK1+ events. A) Representation of samples presented in **Fig. S4-B**: control of non-stained healthy samples cultured in 2D, stained 2D cultures of healthy samples, 2D cultures of ALS samples, and 3D spheroids of healthy samples at different densities, all undergoing *Protocol A*. Violin plot shows HNK1 gated intensity for each condition and their significant differences (one-way ANOVA with Tukey's multiple comparison test, **** p-value<0.0001). Red line represents mean value, dash lines represent lower and upper quartile. **B)** Representation of samples presented in **Fig. S4-C**: control of non-stained healthy spheroids, stained healthy spheroids undergoing protocols A' and D (without CHIR99021), healthy spheroids undergoing protocols B and C (with CHIR99021), ALS spheroids undergoing *protocols A' and D* (without CHIR99021), and ALS spheroids undergoing *protocols B and C* (with CHIR99021). Violin plot shows HNK1 gated intensity for each condition and their significant differences (one-way ANOVA with Tukey's multiple comparison test, **** p-value<0.0001; ** p-value<0.001). Red line represents mean value, dash lines represent lower and upper quartile.

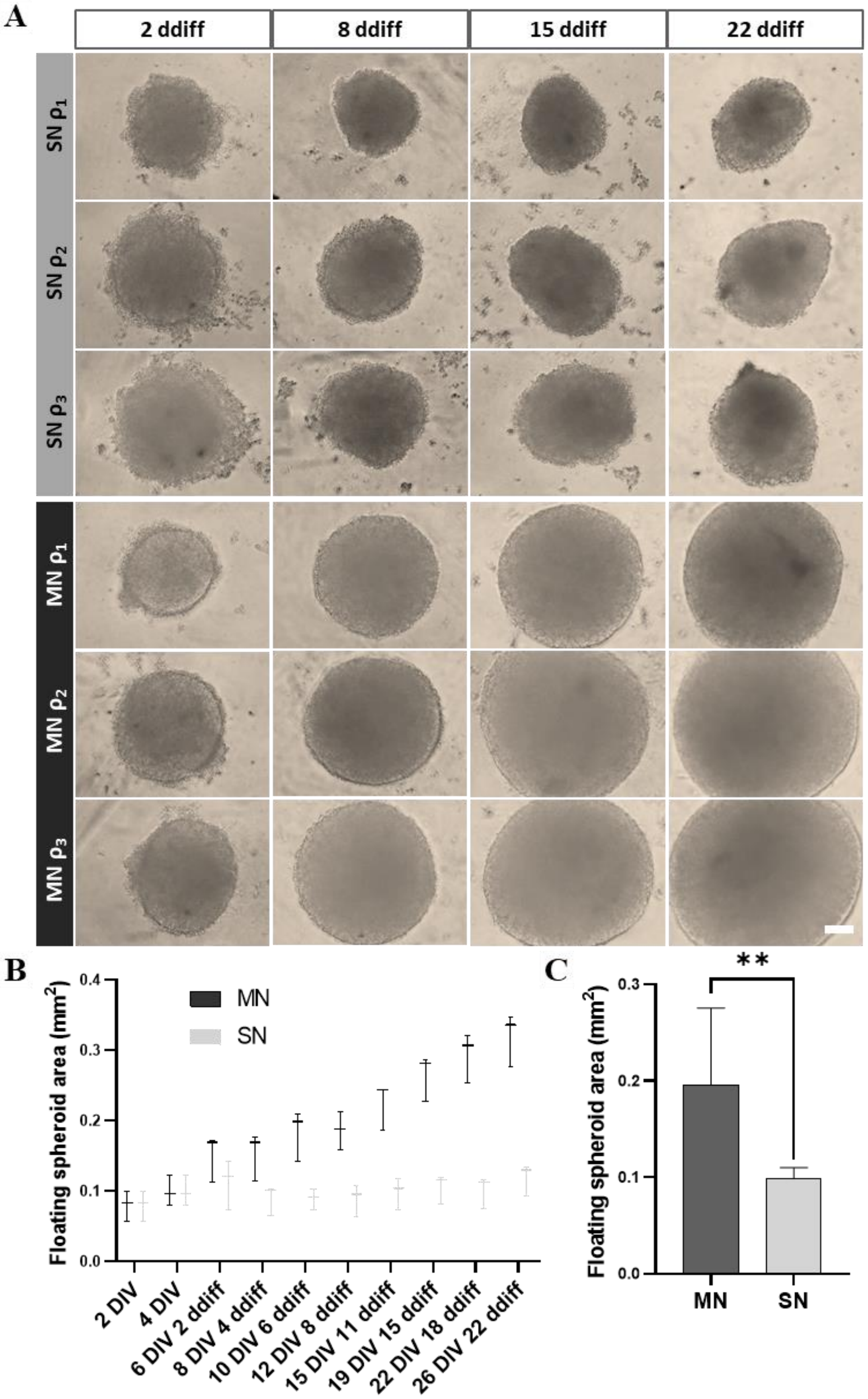

Fig. S6. Comparison of hNSC healthy spheroids differentiated towards pSN and MN as floating spheroids. Timepoints are indicated referring to the days of differentiation (ddiff). **A)** Bright field images of floating spheroids of three different initial seeding densities of hNSC (3.000, 4.500 and 6.000 $\mathrm{cell} \cdot \mathrm{spheroid}^{-1}$) undergoing pSN differentiation protocol A or MN differentiation protocol. Images taken at different days after differentiation started (ddiff). Scale bar 100 µm. **B)** Quantification of spheroid area of floating spheroid images at different timepoints. The box and whiskers plots represent the median values and interquartile ranges for measurements of the three seeding densities obtained for MN and SN spheroids; whiskers represent maximum and minimum values obtained; (n=3, Mann-Whitney test). **C)** Analysis of all measurements grouped obtained for MN and SN

spheroids; bars represent mean ±SD of all values obtained; t- test for unpaired samples with Welch's correction (not assuming equal SDs) resulting in a p-value of 0.0038, **p-value<0.01.

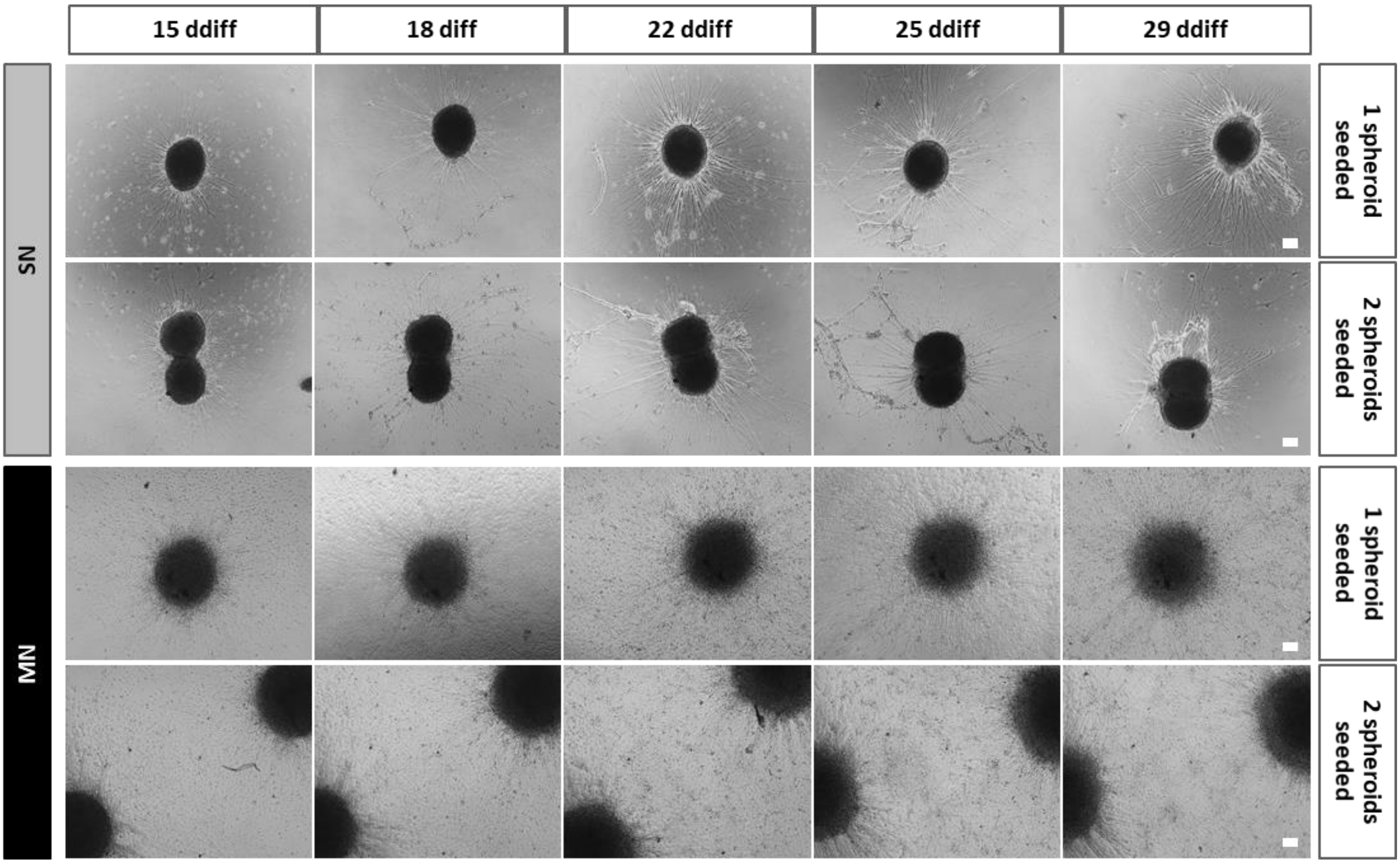

Fig. S7. Comparison of hNSC healthy spheroids differentiated towards pSN and MN as platted spheroids. Timepoints are indicated referring to the days of differentiation (ddiff). Differentiation of healthy hNSC as individual or adjacent plated spheroids. Bright field images of the differentiation of hNSC to SN through differentiation *protocol A* or to MN. Images taken at different timepoints (n=2). Scale bar 100 µm.

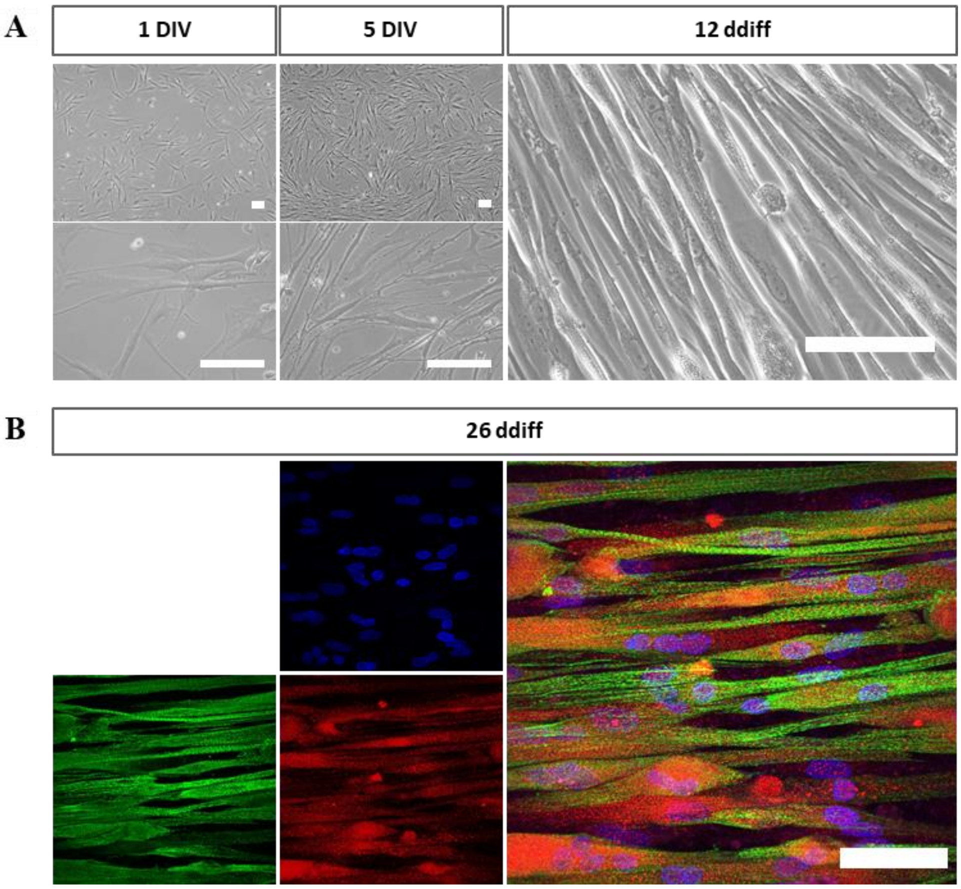

Fig. S8. Proliferation of human skeletal myoblasts and differentiation into myocytes. A) BF images show cell confluency and morphology during the proliferation after 1 DIV, after 5 DIV, and after 12 ddiff. Scale bars 100 µm. **B)** Immunostaining of skeletal myocytes at TP3, after 26 ddiff. Nuclei are stained in blue, MHC in red and α-actinin in green. Scale bar 25 µm.

A

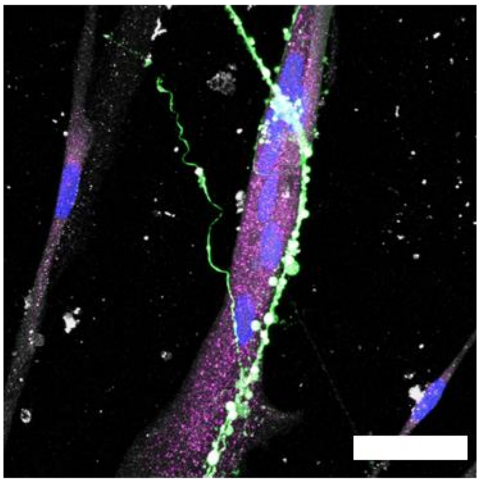

B

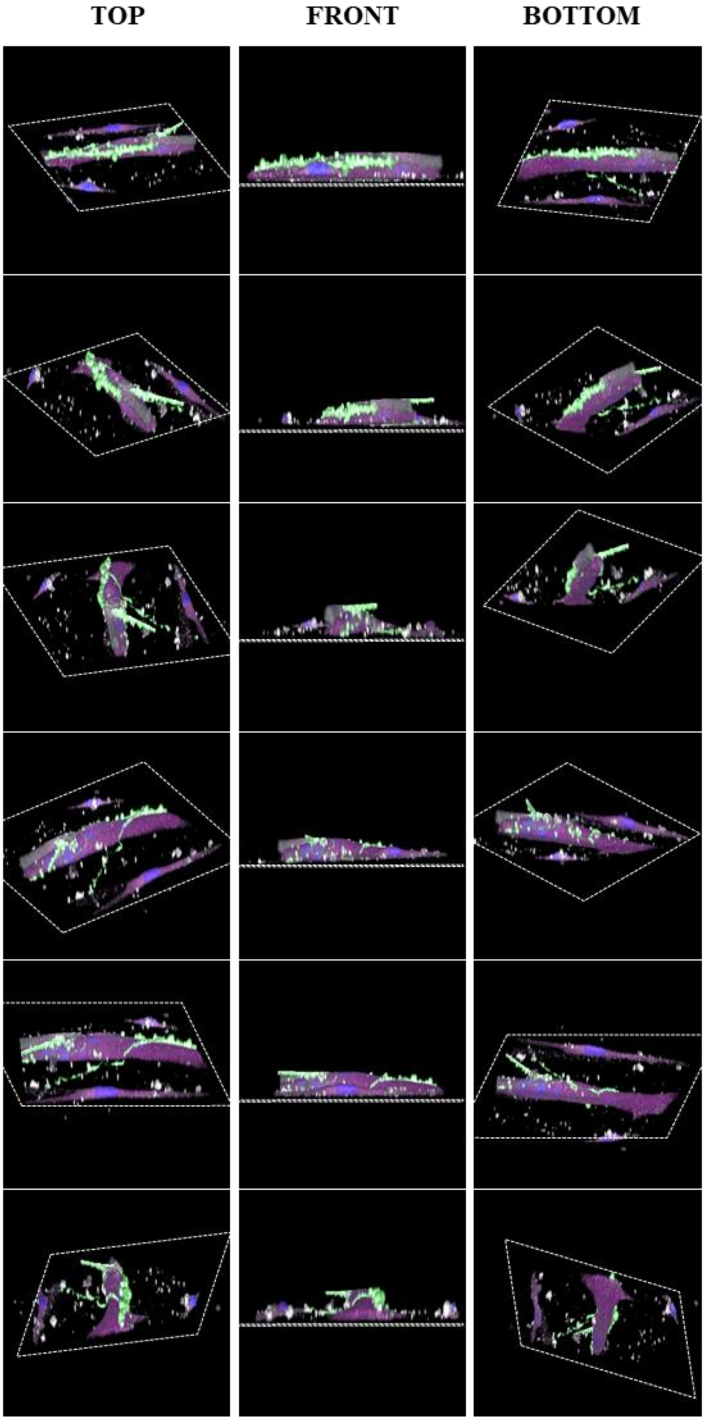

Fig. S9. 3D view rotation of the immunostaining of SkM fibres and SN neurites interaction shown in Figure 6-E.
A) Figure 6-E showing nuclei are shown blue, TUJ1 in green, phalloidin in grey and TrkC in magenta. Scale bar is 25 µm. **B)** 3D view rotation of top, front and bottom perspectives.

Sample of study	Highlights	Ref.
Rat primary sensory neurons cocultured with rat primary myocytes	Immunofluorescence analysis revealed the presence of annulospiral wrapping (ASW) and flower spray ending (FSE), together with the expression in sensory terminals of the stretch sensitive sodium channel BNaC1 and the membrane support protein PICK1.<br>Calcium currents imaging after stretching an intrafusal muscle fibre through microelectromechanical systems, cantilever deflection, detected the presence of physiologically relevant sensory endings.	Rumsey *et al.*, 2010 [48]
Rat dissociated DRG cells cocultured with rat primary skeletal muscle cells	Observation through immunofluorescence, phase contrast imaging and scanning electron microscopy (SEM) of the interrelation and contacts formed between sensory neurons and skeletal muscle cells.	Liu *et al.,* 2011 [49]
Human neural progenitors derived sensory neurons cocultured with human skeletal muscle stem cells derived intrafusal fibres	They observed bag and chain intrafusal fibre morphologies through phase contrast imaging and FSE and ASW morphologies through immunofluorescence imaging. Patch-clamp electrophysiological recordings suggest that human intrafusal muscle fibres have a repetitive firing rate, in contrast to human extrafusal fibres.	Guo *et al.*, 2017 [43]
Rat DRG explants cocultured with skeletal muscle cells dissociated from rat limb	NRG-1β treatment promoted neuronal migration from the DRG explants and neurite outgrowth. This modulation, correlated with GAP-43 expression, could be linked to intrafusal muscle fibre formation.	Qiao *et al.,* 2018 [50]

Table S1. Summary of the publications studying the sensory afferent pathway *in vitro* in the last decade though SN-SkM coculture. The cells utilised and highlighted results are described for each publication reference.

Gene	Encoding protein and function
GAPDH	Glyceraldehyde 3-phosphate dehydrogenase plays a role in cell glycolysis and nuclear functions. It is implicated in metabolic and non-metabolic functions, such as initiation of apoptosis or axoplasmic transport. It is frequently used as a housekeeper gene.
ACTB	β-actin, a human isoform of actin, is a highly preserved protein involved in cell structure, integrity and motility. It is frequently used as a housekeeper gene.
RPS18	Ribosomal protein S18 is a component of the 40S subunit of ribosomes, organelles that catalyse protein synthesis. It is frequently used as a housekeeper gene.
HNK1	Galactosylgalactosylxylosylprotein 3-β-glucuronosyltransferase 1 (B3GAT1) is an enzyme that in humans is encoded by the B3GAT1 gene, also known in immunology as HNK1 (human natural killer-1). It is involved in cell metabolism and expressed in NCSC.[46,51]
TUBB3	Class III β-tubulin is a microtubule element of the tubulin family, found almost exclusively in neurons and testis. It plays a role in DRG axon projection towards the spinal cord.[92]
PRPH	Peripherin is a type III intermediate filament protein expressed mainly in neurons of the PNS and motor neurons.[38]<br>It is known to be upregulated in ALS.[56]
POU4F1	POU domain, class 4, transcription factor 1 (POU4F1) also known as brain-specific homeobox/POU domain protein 3A (BRN3A), is a protein highly expressed in the developing peripheral sensory nervous system (e.g. DRG). [35,38,93]
NTRK1	Tropomyosin receptor kinase A (TrkA), also known as neurotrophic tyrosine kinase receptor type 1 (NTRK1), is expressed in peptidergic and some nonpeptidergic nociceptive neurons, and binds to nerve growth factor (NGF).[37,61]
NTRK2	Tropomyosin receptor kinase B (TrkB), also known as neurotrophic tyrosine kinase receptor type 2 (NTRK2) is expressed in some mechanoreceptive type of sensory neurons and binds to brain-derived neurotrophic factor (BDNF).[37,61]
NTRK3	Tropomyosin receptor kinase C (TrkC), also known as neurotrophic tyrosine kinase receptor type 3 (NTRK3), is expressed in proprioceptive type of sensory neurons and some mechanoreceptive neurons.[61] It binds to neurotrophin-3 (NT-3), mediating neuronal differentiation and survival.[37,94]
RUNX3	Runt-related transcription factor 3 (Runx3) regulates survival and axonal projections of proprioceptive sensory neurons.[60] Runx3 has a different regulation in each subtype of TrkC+ neuron.[67]
DICER	Dicer is essential for maintenance rather than initiation of synaptic contacts in sensorimotor connections, and for processing micro RNAs (miRNAs). Selective impairment in sensory neurons causes sensory ataxia.[59,60]
ETV1	ETS variant 1 expression is induced by NT3-TrkC signalling and it is involved in proprioceptive axon projection regulation.[60,66] It is also needed for the survival and differentiation of pSN.[95] But it is also critical in the formation of functional connections between pSN and MN.[66]
SPP1	Secreted phosphoprotein 1 is important for cell-matrix interaction and immune functions, and is known to be expressed in pSN.[61] It is frequently present in the inflammatory environment of dystrophic and injured muscles.[61,62]
VGluT1	Vesicular glutamate transport 1 (VGluT1), also known as solute carrier family 17 member 7 (SLC17A7), transports glutamate to synaptic vesicles before exocytotic release. It is expressed in pSN at the site of innervation of intrafusal fibers.[96] But it is also detected at the synaptic contacts of interneurons coming from the spinal cord and

	descending cortical axons, being expressed in the spinal cord in the dorsal horn, intermediate gray and ventral horn, and in sensory DRG neurons.[26]
PVALB	Parvalbumin, although frequently used as a proprioceptive sensory neuron marker, is also expressed in other cells of the motor system, including but not limited to motoneurons and interneurons within the spinal cord and muscles. [59,65,66]
MBP	Myelin basic protein is the major constituent of the myelin sheath formed by oligodendrocytes, in the CNS, and Schwann cells, in the PNS. Both MN and pSN are myelinated cells.[61] Anormal levels of MBP are related with demyelinating diseases, multiple sclerosis and ALS. Myelin sheath ultrastructure is known to be disorganized in ALS.[97] Furthermore, oligodendrocyte maturation and MBP expression is known to be reduced in ALS,[10,11] and myelinating Schwann cells are known to be damaged in ALS and trigger inflammatory mechanisms in ALS peripheral nerves.[5,6]
GFAP	Glial fibrillary acidic protein is one of the major filament proteins of mature astrocytes. It is also present in Schwann cells under stress conditions and following nerve damage or denervation, and it has been found to be upregulated in the peripheral nerve Schwann cells in ALS.[57]
MNX1	Motor neuron and pancreas homebox 1, also known as homeobox HB9 (HLXB9), used for identification of MN, it is involved in neural stem cell differentiation pathway and lineage-specific markers.
TNFSF1B	Tumour necrosis factor receptor superfamily member 1B, also known as p75 tumour necrosis factor receptor (P75TNFR), mediates anti-apoptotic and inflammation signals, and it is related with ALS pathway as it is observed in higher expression levels in ALS patients.[58]

Table S2. Genes analysed in the qPCR: gene name. encoding protein and function. The information for gene description is taken from the databases Genecards[98] and OMIM,[99] and other papers cited below.

Medium	Components and final concentration	Commercial reference
hNSC proliferation medium	Knockout™ DMEM/F-12	(Gibco, #12660012)
	1X final concentration of StemPro™ neural supplement	(Gibco, #A1050801)
	2 mM of GlutaMAX™ supplement	(Gibco, #35050061)
	20 $ng \cdot ml^{-1}$ of FGF-basic (aminoacid 10-155) recombinant human protein	(Gibco, #PHG0024)
	20 $ng \cdot ml^{-1}$ of EGF recombinant human protein	(Gibco, #PHG0314)
Medium hSN2A	Knockout™ DMEM/F-12	(Gibco, #12660012)
	1X final concentration of StemPro™ neural supplement	(Gibco, #A1050801)
	2 mM of GlutaMAX™ supplement	(Gibco, #35050061)
	500 $ng \cdot ml^{-1}$ of recombinant human Noggin	(Goldbio, #1180-09-20)
	10 μM of SB431542	(StemCell Technologies, #72232)
	(*) 3 μM of CHIR99021	(Caymanchem, #13122)
Medium hSN2B	Neurobasal™ Plus medium	(Gibco, #A3582901)
	1X final concentration of B-27™ Plus supplement	(Gibco, #A3582801)
	10 $ng \cdot ml^{-1}$ of recombinant human BDNF	(R&D Systems, #248-BD-025)
	10 $ng \cdot ml^{-1}$ of recombinant human GDNF	(R&D Systems, #212-GD-010)
	10 $ng \cdot ml^{-1}$ of recombinant human NGF	(Axol Bioscience, #ax139789)
	10 $ng \cdot ml^{-1}$ of human Wnt-1	(Axol Bioscience, #ax135565)
	10 $ng \cdot ml^{-1}$ of human NT-3	(Axol Bioscience, #ax139811)
	10 μM of 8-Br-cAMP	(Axxora, #BLG-B007)
	200 μM of L-Ascorbic acid	(Sigma-Aldrich, #A4403)
	500 $ng \cdot ml^{-1}$ of recombinant human Noggin	(Goldbio, # 1180-09-20)
	10 μM of SB431542	(StemCell Technologies, #72232)
	(*) 3 μM of CHIR99021	(Caymanchem, #13122)
Medium hSN3	Neurobasal™ Plus medium	(Gibco, #A3582901)
	1X final concentration of B-27™ Plus supplement	(Gibco, #A3582801)
	10 $ng \cdot ml^{-1}$ of recombinant human BDNF	(R&D Systems, #248-BD-025)
	10 $ng \cdot ml^{-1}$ of recombinant human GDNF	(R&D Systems, #212-GD-010)
	10 $ng \cdot ml^{-1}$ of recombinant human NGF	(Axol Bioscience, #ax139789)
	10 $ng \cdot ml^{-1}$ of human Wnt-1	(Axol Bioscience, #ax135565)
	10 μM of 8-Br-cAMP	(Axxora, #BLG-B007)
	200 μM of L-Ascorbic acid	(Sigma-Aldrich, #A4403)
	(**) $10-50$ $ng \cdot ml^{-1}$ of human NT-3	(Axol Bioscience, #ax139811)

	(*) 25 μM of ROCK inhibitor Y27632	(R&D Systems, #1254)
hSkMb proliferation medium	SKBM™-2 Skeletal Muscle Cell Growth Basal Medium-2	(Lonza, #CC-3246)
	hEGF	(Lonza, #CC-3244)
	Dexamethasone	(Lonza, #CC-3244)
	L-glutamine	(Lonza, #CC-3244)
	FBS	(Lonza, #CC-3244)
	Gentamicin / Amphotericin-B	(Lonza, #CC-3244)
hSkMc differentiation medium	DMEM, high glucose, pyruvate	(Gibco, #11995-065)
	Horse serum (***)	(Gibco, #16050130)
	1% penicillin streptomycin	(Gibco, #15140122)
	10 $ng \cdot ml^{-1}$ hEGF	(Gibco, #PHG0311)
	50 $ng \cdot ml^{-1}$ LONG® R3 IGF-I human	(Sigma Aldrich, #I1271)
hMN medium	DMEM/F-12, GlutaMAX™ supplement	(Gibco, #10565-018)
	1X final concentration of StemPro® hESC Supplement	(Gibco, #A10006-01)
	2% BSA	(Gibco, #A10008-01)
	200 $ng \cdot ml^{-1}$ of recombinant human Shh	(Peprotech, #100-45)
	50 μM of retinoic acid	(Sigma-Aldrich, #R2625)
	10 $ng \cdot ml^{-1}$ of recombinant human, murine, rat Activin A (E.coli derived)	(Peprotech, #120-14E)
	8 $ng \cdot ml^{-1}$ of recombinant human FGF basic (154 aminoacids)	(Peprotech, #100-18B)
	10 $ng \cdot ml^{-1}$ of human recombinant BDNF, animal component free	(StemCell Technologies, #78133)
	10 $ng \cdot ml^{-1}$ of recombinant human GDNF	(R&D Systems, #212-GD-010)

Table S3. Composition of the media used for these experiments. hNSC = human neural stem cell; FGF = fibroblast growth factor; EGF = epidermal growth factor; NCSC = neural crest stem cell; NGF = nerve growth factor; GDNF = glial cell line-derived neurotrophic factor; BDNF = brain-derived neurotrophic factor; NT-3 = neurotrophin-3; 8-Br-cAMP = 8-Bromoadenosine-3',5'-cyclic monophosphate; hSkMb = human skeletal myoblasts; hSkMc = human skeletal myocytes; hEGF = human epidermal growth factor; FBS = foetal bovine serum; IGF-I = insulin like growth factor type I; hESC = human embryonic stem cell; BSA = bovine serum albumin; Shh = Sonic Hedgehog; E.coli = Escherichia coli. (*) = only in some conditions. (**) = varying concentration for each protocol tried. (***) = varying concentration between 2% and 10% at different stages.

	**pSN differentiation protocol**				
	**Protocol A**	**Protocol A'**	**Protocol B**	**Protocol C**	**Protocol D**
**Medium SN2A and SN2B (TP1 to TP2)**	-	-	+ CHIR99021	+ CHIR99021	-
***Medium SN3* (TP2 to TP3)**	+ NT-3 $10\ \mathrm{ng \cdot ml^{-1}}$	+ NT-3 $50\ \mathrm{ng \cdot ml^{-1}}$	+ NT-3 $50\ \mathrm{ng \cdot ml^{-1}}$	*+ ROCK inhibitor Y27632*<br>+ NT-3 $50\ \mathrm{ng \cdot ml^{-1}}$	*+ ROCK inhibitor Y27632*<br>+ NT-3 $50\ \mathrm{ng \cdot ml^{-1}}$

Table S4. Overview of the main differences among the proprioceptive sensory neuron differentiation protocols performed. TP1 = timepoint 1; TP2 = timepoint 2; TP3 = timepoint 3; NT-3 = neurotrophin-3.

Target gene	Gene ID	Forward primer sequence	Reverse primer sequence
GAPDH	2597	GGAGCGAGATCCCTCCAAAAT	GGCTGTTGTCATACTTCTCATGG
ACTB	60	CATGTACGTTGCTATCCAGGC	CTCCTTAATGTCACGCACGAT
RPS18	6222	GCGGCGGAAAATAGCCTTTG	GATCACACGTTCCACCTCATC
HNK1 (a.k.a. B3GAT1)	27087	CTCCTTCGAGAACTTGTCACC	GGGTCAGTGAAGCCCTTCTT
TUBB3	10381	GGCCAAGGGTCACTACACG	GCAGTCGCAGTTTTCACACTC
PRPH	5630	GCCTGGAACTAGAGCGCAAG	CCTCGCACGTTAGACTCTGG
POU4F1 (a.k.a. BRN3A)	5457	GGGCAAGAGCCATCCTTTCAA	CTGTTCATCGTGTGGTACGTG
NTRK1 (a.k.a. TrkA)	4914	AACCTCACCATCGTGAAGAGT	TGAAGGAGAGATTCAGGCGAC
NTRK2 (a.k.a. TrkB)	4915	TCGTGGCATTTCCGAGATTGG	TCGTCAGTTTGTTTCGGGTAAA
NTRK3 (a.k.a. NTRK3)	4916	ACGAGAGGGTGACAATGCTG	CCAGTGACTATCCAGTCCACA
RUNX3	864	AGGCAATGACGAGAACTACTCC	CGAAGGTCGTTGAACCTGG
DICER	23405	TGCTATGTCGCCTTGAATGTT	AATTTCTCGATAGGGGTGGTCTA
ETV1 (a.k.a. ER81)	2115	TGGCAGTTTTTGGTAGCTCTTC	CGGAGTGAACGGCTAAGTTTATC
SPP1 (a.k.a. OPN)	6696	CTCCATTGACTCGAACGACTC	CAGGTCTGCGAAACTTCTTAGAT
VGluT1 (a.k.a. SLC17A7)	57030	CGACGACAGCCTTTTGTGGT	GCCGTAGACGTAGAAAACAGAG
PVALB	5816	AAGAGTGCGGATGATGTGAAG	GCCTTTTAGGATGAATCCCAGC
MBP	4155	CACGCAGGCAAACGAGAATTA	CTGAGGTTGTCCGTGAAAGTT
GFAP	2670	CTGCGGCTCGATCAACTCA	TCCAGCGACTCAATCTTCCTC
MNX1 (a.k.a. Hb9)	3110	GATGCCCGACTTCAACTCCC	GCCGCGACAGGTACTTGTT
TNFRSF1B (a.k.a. P75TNFR)	7133	CGGGCCAACATGCAAAAGTC	CAGATGCGGTTCTGTTCCC

Table S5. qPCR primers. Target gene name, with alternative names, Gene ID code and forward and reverse sequences (both 5’-3’) are indicated. All gene sequences are for *Homo sapiens*.

Movie S1. 3D reconstruction of the immunostaining of an interaction between TrkC+ neuron and a muscle fibre. The video shows a 3D reconstruction of the **Figure 6-E** rotating. Nuclei are shown in blue, TUJ1 in green, phalloidin in grey and TrkC in magenta.

Movie S2. 3D reconstruction of the immunostaining of an interaction between TrkC+ neuron and a muscle fibre. The video shows a 3D reconstruction of the **Figure 6-E** rotating in the Y axis. Nuclei are shown in blue, TUJ1 in green, phalloidin in grey and TrkC in magenta.
